# Caspase Inhibition Modulates Monocyte-Derived Macrophage Polarization in Damaged Tissues

**DOI:** 10.3390/ijms24044151

**Published:** 2023-02-19

**Authors:** Stéphanie Solier, Michele Mondini, Lydia Meziani, Arnaud Jacquel, Catherine Lacout, Tom Vanden Berghe, Yvon Julé, Jean-Claude Martinou, Gérard Pierron, Julie Rivière, Marc Deloger, Corinne Dupuy, Anny Slama-Schwok, Nathalie Droin, Peter Vandenabeele, Patrick Auberger, Eric Deutsch, Jamel El-Benna, Pham My-Chan Dang, Eric Solary

**Affiliations:** 1 INSERM U1170, Gustave Roussy, 94805 Villejuif, France; 2 Faculty of Medicine, Université Paris-Sud, 94270 Le Kremlin-Bicêtre, France; 3 Laboratoire d’Excellence LipSTIC, 21000 Dijon, France; 4 INSERM UMR1030, Gustave Roussy, 94805 Villejuif, France; 5 INSERM U1065/C3M, 06204 Nice, France; 6 Inflammation Research Center (IRC), VIB, 9052 Ghent, Belgium; 7 Department of Biomedical Molecular Biology, University of Ghent, 9000 Ghent, Belgium; 8 Biocellvia, 13001 Marseille, France; 9 Department of Cell Biology, University of Geneva, 1211 Geneva, Switzerland; 10 CNRS UMR8122, Gustave Roussy, Villejuif, 94805, France; 11 AMMICa, INSERM US23, CNRS UMS 3655, Gustave Roussy, 94805 Villejuif, France; 12 CNRS UMR8200, Gustave Roussy, 94805 Villejuif, France; 13 INSERM U1287, Gustave Roussy, 94805 Villejuif, France; 14 Centre de Recherche sur l’Inflammation (CRI), Laboratoire d’Excellence Inflamex, Faculté de Médecine Xavier Bichat, Université de Paris, INSERM-U1149, CNRS-ERL8252, 75018 Paris, France

**Keywords:** caspase, CSF1, differentiation, macrophage, NOX2, lung fibrosis

## Abstract

Circulating monocytes are recruited in damaged tissues to generate macrophages that modulate disease progression. Colony-stimulating factor-1 (CSF-1) promotes the generation of monocyte-derived macrophages, which involves caspase activation. Here, we demonstrate that activated caspase-3 and caspase-7 are located to the vicinity of the mitochondria in CSF1-treated human monocytes. Active caspase-7 cleaves p47*^PHOX^* at aspartate 34, which promotes the formation of the NADPH (nicotinamide adenine dinucleotide phosphate) oxidase complex NOX2 and the production of cytosolic superoxide anions. Monocyte response to CSF-1 is altered in patients with a chronic granulomatous disease, which are constitutively defective in NOX2. Both caspase-7 down-regulation and radical oxygen species scavenging decrease the migration of CSF-1-induced macrophages. Inhibition or deletion of caspases prevents the development of lung fibrosis in mice exposed to bleomycin. Altogether, a non-conventional pathway that involves caspases and activates NOX2 is involved in CSF1-driven monocyte differentiation and could be therapeutically targeted to modulate macrophage polarization in damaged tissues.

## 1. Introduction

Macrophages are phagocytic cells that exert homeostatic functions in every tissue. Resident macrophages either arise from yolk sac progenitors [1] or are continuously replaced through recruitment and differentiation of fetal liver- or bone-marrow-derived monocytes [2]. In damaged tissues, recruited macrophages mostly derive from circulating monocytes [3]. These plastic cells initially show a pro-inflammatory phenotype, then undergo apoptosis or switch to an anti-inflammatory phenotype that facilitates tissue repair [4]. This switch has a major role in chronic diseases such as cancer, autoimmune and inflammatory diseases, and tissue fibrosis [5,6,7]. 

The recruitment and functions of macrophages in damaged tissues critically depend on colony-stimulating factor-1 (CSF1). CSF1 receptor (CSF1R) blockade with either antibodies or small molecules has been tested in various diseases [8,9,10]. However, resistance to CSF1R blockade occurs, e.g., through modification of a tumor microenvironment [11]. Therefore, there is a need for alternative strategies to modulate macrophage activity in various diseases. 

CSF1-induced differentiation of monocytes into macrophages [12] is associated with activation of several caspases in the absence of cell death [13,14]. In peripheral blood monocytes, CSF1 interaction with CSF1R at the cell surface generates intracellular waves of phosphatidylinositol-3-kinase activation and AKT phosphorylation that induce the formation of a multiprotein complex [15]. Formed in the absence of death receptor, this complex associates procaspase-8 to the adaptor molecule FADD (Fas-Associated Death Domain), the serine-threonine kinase RIPK1 (Receptor Interacting Protein Kinase 1) and the adaptor molecule FLIP_L_ (cellular FLICE-like inhibitory protein long form) [16]. In turn, caspase-8 is activated and activates caspase-3 and caspase-7 that cleave intracellular proteins such as nucleophosmin (NPM1) [17,18]. It remains unclear why the activation of these enzymes in response to CSF-1 does not kill monocytes, and to what extent active caspases contribute to the generation of full-blown functional macrophages.

Here, we use primary human monocytes and mouse models of caspase deletion in myelomonocytic cells to further explore the role of these enzymes in CSF1-mediated generation of macrophages. We show that active caspase-3 and caspase-7 are spatially restricted to the surface of the mitochondria. p47*^PHOX^*, a subunit of the NADPH (nicotinamide adenine dinucleotide phosphate) oxidase NOX2, is cleaved by active caspase-7, which promotes NOX2 activation and the generation of intracellular reactive oxygen species (ROS), a process required for full differentiation. Inhibition of this caspase-7/NOX2 axis alters the migration of CSF1-induced macrophages.

To explore caspase inhibition effects in vivo, we used a lung fibrosis mouse model, more specifically bleomycin-induced lung fibrosis, as multiple studies indicated a central role of macrophages in this disease [6,19,20,21,22,23]. We report that monocyte-restricted deletion of upstream caspase-8 and treatment with the clinically relevant pan-caspase inhibitor IDN-6556 prevent bleomycin-induced lung fibrosis in mice, indicating caspase inhibition as an alternative strategy to CSF1R blockade to modulate macrophage polarization in damaged tissues.

## 2. Results

### 2.1. Caspases Modulate CSF1-Induced Monocyte Differentiation

Exposure of monocytes to CSF1 or to granulocyte–macrophage colony-stimulating factor (GM-CSF) induces their ex vivo differentiation into macrophages [24]. DEVD and LETD cleavage activities (which correspond to caspases-3/7 activity and caspase-8 activity, respectively) appear in human monocytes cultured in the presence of CSF1, not in those cultured with GM-CSF (Figure S1A). After five days, CSF1-treated monocytes acquire a “fibroblast-like” elongated shape (Figure S1B,C) with a CD14^+^, CD16^+^, CD71^+^, CD163^+^ phenotype (Figure S1D–F). These changes are prevented by co-culture with the broad spectrum caspase inhibitor Q-VD-OPh (Quinoline-Val-Asp-Difluorophenoxymethylketone) (Figure S1B–F) while the more specific inflammatory caspase inhibitor Ac-YVAD-cmk (N-acetyl-tyrosyl-valyl-alanyl-aspartyl chloromethyl ketone) had no significant impact (Figure S1C,F). The phenotype of macrophages generated from CSF1 + Q-VD-OPh-treated monocytes was partly reminiscent of that observed in GM-CSF-treated monocytes (Figure S1C,F). Q-VD-OPh also interfered with CSF1-induced differentiation of mouse monocytes, i.e., the pan-caspase inhibitor prevented acquisition of a CD206^+^, CD71^+^ phenotype while promoting the expression of cell surface markers CD40 and CD54 (Figure S1G).

**Figure 1:**
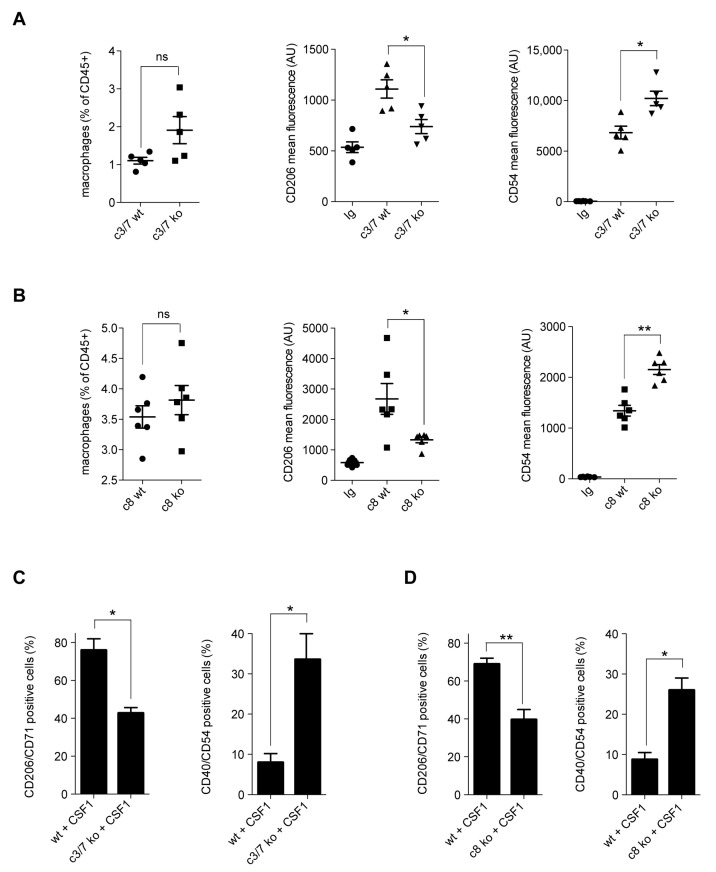
Macrophage alterations in monocyte-restricted caspase knockout mice. (**A**,**B**) Interstitial macrophages were sorted from the lung of monocyte-restricted caspase-3/7 double knockout (LysM-Cre/Casp3/7^fl/fl^) or caspase-8 knockout (LysM-Cre; Casp8^fl/fl^) mice or wild-type (wt) animals. Interstitial macrophages were selected according to their larger size (FSC) and granularity (SSC) as CD45^+^, GR1^−^, CD11b^high^, SiglecF^−^, IA-IE^+^, CD24^−^ cells. (**A**) Left panel, number of interstitial macrophages, expressed as percent of CD45^+^ cells, in wild-type and LysM-Cre/Casp3/7^fl/fl^ mice (c3/7 ko). Flow cytometry analysis of cell surface markers CD206 (middle panel) and CD54 (right panel) expressed as mean fluorescence intensity (*, *p* < 0.05; ns, non-significant; Mann–Whitney test, *n* = 5 per group). Mean ± SE. (**B**) Left panel, number of interstitial macrophages, expressed as percent of CD45^+^ cells, in wild-type and LysM-Cre; Casp8^fl/fl^ mice (c8 ko). Flow cytometry analysis of cell surface markers CD206 (middle panel) and CD54 (right panel) expressed as mean fluorescence intensity (*, *p* < 0.05; **, *p* < 0.01; ns, non-significant; Mann–Whitney test, *n* = 6 per group). Mean ± SE. (**C**,**D**) Bone marrow cells were flushed from mouse femurs and tibias of LysM-Cre/Casp3/7^fl/fl^ (**C**) or LysM-Cre; Casp8^fl/fl^ mice (**D**) or wild-type (wt) animals before sorting CD115^+^/CD11c^−^/NK^−^ monocytes. These cells were cultured in the presence of 100 ng/mL CSF1 for 5 days before multiparameter flow cytometry analysis. Left panels show the percentage of CD206^+^/CD71^+^ macrophages. Right panels indicate the percentage of CD40^+^/CD54^+^ macrophages (*, *p* < 0.05; **, *p* < 0.01; Mann–Whitney test, *n* = 4). Mean ± SE.

To gain further insights into the role of caspases in CSF1-induced differentiation of monocytes, we used monocyte-restricted caspase-8 knockout (LysM-Cre; Casp8^fl/fl^) and caspase 3/caspase-7 double knockout (LysM-Cre; Casp3/7^fl/fl^) mice (Figure S2A,B). The number of lung interstitial macrophages remained unchanged compared to wild-type mice but these cells expressed less CD206 and more CD54 at their surface (Figure 1A,B and Figure S2C). Of note, the number of blood monocytes is similar in knockout and wild-type animals. LysM driven deletion of caspase-3 and caspase-7, as well as that of caspase-8, prevented CSF1-treated monocytes from acquiring a CD206^+^/CD71^+^ phenotype and promoted acquisition of a CD40^+^/CD54^+^ phenotype (Figure 1C,D). These results indicate a role for caspases in the phenotype of macrophages induced by CSF1-triggered differentiation of monocytes, both in vivo and ex vivo.

### 2.2. Active Caspase-3 and Caspase-7 Are Spatially Restricted to the Mitochondria Outer Membrane

**Figure 2:**
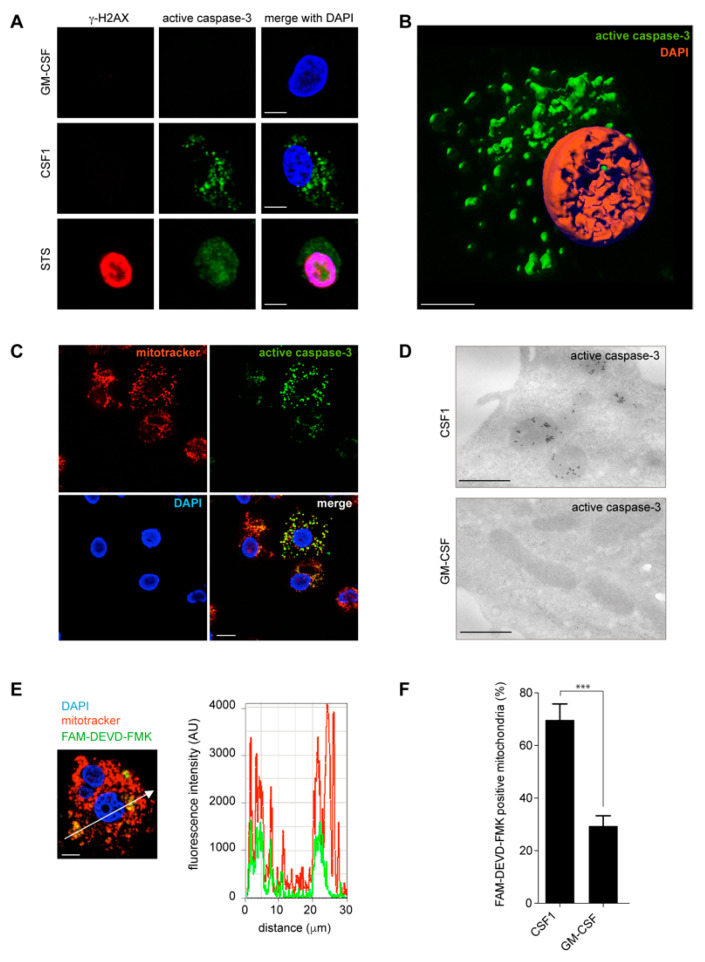
Active caspase-3 and caspase-7 are located to the mitochondria in CSF-1 treated human monocytes (**A**) Active caspase-3 (green), γ-H2AX (red) and DAPI (blue) staining of peripheral blood human monocytes treated with GM-CSF (100 ng/mL, 4 days) or CSF-1 (100 ng/mL, 4 days) or staurosporine (STS, 1 µM, 3 h). Scale bar, 5 µm. (**B**) 3D single-cell analysis of active caspase-3 (green) in CSF-1-treated monocyte (nucleus in red, DAPI staining). Scale bar, 5 µm. (**C**) Active caspase-3 (green), mitochondria (mitotracker, red) and nucleus (DAPI, blue) staining in CSF-1-treated monocytes. Scale bar, 10 µm. Threshold set to 300 (green) and 800 (red) for co-localization analyses with ImageJ-JACoP; Manders coefficient: 0.812 (fraction of active caspase-3 co-localized with mitochondria). (**D**) Immunogold staining of active caspase-3 in CSF-1- and GM-CSF-treated monocytes showing the co-localization of mitochondria and active caspase-3 in CSF-1-treated monocytes. Scale bar, 0.5 µm. (**E**) Confocal microscopy image (left panel) and intensity tracing (right panel) of FAM-DEVD-FMK (green) and mitotracker (red) distribution in a CSF-1-treated monocyte (DAPI staining of the nucleus in blue). Scale bar, 5 µm. (**F**) FAM-DEVD-FMK intensity measured by flow cytometry in tetramethylrhodamine (TMRM) positive mitochondria isolated from CSF-1 and GM-CSF-treated human monocytes (***, *p* < 0.001; Fisher Exact Test). Mean ± SE.

To explore where and how caspases were activated in CSF1-treated monocytes, we used fluorescence microscopy and focused initially on caspase-3 (Figure 2). Exposure of human monocytes to staurosporine, a non-selective protein kinase inhibitor used as an apoptosis inducer, induced their rapid death by apoptosis with a characteristic γ-H2AX (H2AX, H2A histone family member X) nuclear ring [25] and a diffuse active caspase-3 staining observed in both the nucleus and the cytoplasm (Figure 2A). In contrast, active caspase-3 accumulated in the cytoplasm of CSF1-treated monocytes, in the absence of any γ-H2AX nuclear ring (Figure 2A,B). Such a labeling was prevented by co-treatment of monocytes with the pan-caspase inhibitor Q-VD-OPh (Figure S3A) and its specificity further supported by using a blocking peptide that prevented any staining with the anti-active caspase-3 antibody (Figure S3B). The active protease did not co-localize with LC3, which labels autophagic vesicles (Figure S3C), nor with calreticulin that identifies the endoplasmic reticulum (Figure S3D). Confocal fluorescent images demonstrated that active caspase-3 strikingly co-localized with mitochondria labeled with mitotracker (Figure 2C, Manders’ coefficient: 0.812). Electron microscopy using immunogold staining further demonstrated that active caspase-3 was specifically located at the mitochondria of CSF1-treated monocytes (Figure 2D). Fluorescence microscopy detected the fluorochrome-associated caspase-3 and caspase-7 substrate FAM-DEVD-FMK in the mitochondria of CSF1-treated monocytes by fluorescence microscopy (Figure 2E and Figure S3E), which was confirmed by flow cytometry analysis of mitochondria sorted from CSF1-treated monocytes (Figure 2F). 

**Figure 3:**
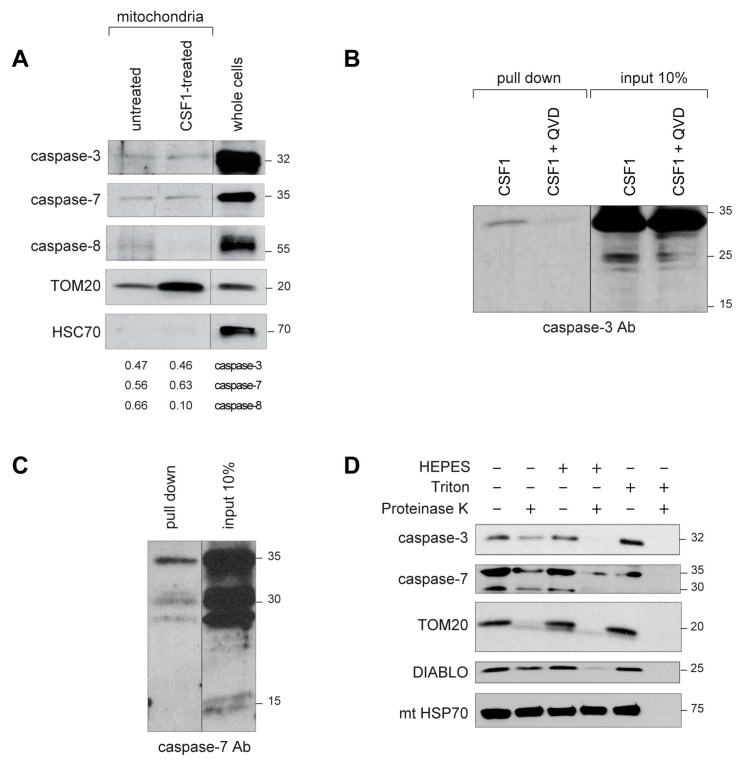
Active forms of caspase-3 and caspase-7 and their localization to the outer mitochondrial membrane. Sorted human monocytes were analyzed before any treatment or after a 4-day exposure to 100 ng/mL CSF-1, with or without 50 µM Q-VD-OPh (**A**) Immunoblot analysis of indicated proteins in lysates of mitochondria sorted from untreated and CSF1-treated monocytes, and in total cell lysates of CSF1-treated cells. (**B**,**C**) In situ caspase trapping assays performed in CSF-1-treated monocytes in the absence or presence of Q-VD-OPh. Inputs (10%) shown for comparison. Immunoblot detection of active caspase-3 (**B**) and active caspase-7 (**C**) in whole cell lysates and in mitochondrial lysates respectively, using anti-caspase-3 and an anti-caspase-7 antibodies. (**D**) Immunoblot analysis of indicated proteins in mitochondria isolated from CSF-1-treated monocytes and exposed to proteinase K or HEPES or Triton X100. Protein ladder (kDa) is indicated.

Immunoblot analysis of caspase-3 and caspase-7 expression in mitochondria isolated from untreated and CSF-1-treated human monocytes demonstrated that the fraction of these enzymes associated to these organelles was low and did not increase upon CSF-1 treatment (Figure 3A). To determine how caspase-3 was activated in the mitochondria of CSF-1-treated human monocytes, we performed in situ caspase trapping using cell lysates and biotinylated DEVD-FMK, demonstrating that the active enzyme was the full-length, 32 kDa caspase-3, this activity being lost in the presence of Q-VD-OPh (Figure 3B). In situ caspase trapping experiments using mitochondrial lysates also detected active caspase-7 in mitochondria, demonstrating that both the full length 35 kDa protein and its ~30 kDa fragment were enzymatically active (Figure 3C). To determine more precisely the localization of these enzymes at the mitochondria level, we isolated these organelles from CSF-1-treated monocytes and treated them with proteinase K, with or without detergents of increasing activity (Figure 3D). The expression of the 32 kDa caspase-3, as well as that of the 35 kDa caspase-7 and its ~30 kDa fragment, decreased in mitochondria treated with proteinase K, which eliminated proteins associated with the outer membrane of these organelles such as TOM20. Proteins of the inter-membrane space such as DIABLO (direct IAP binding protein with low pI) disappeared when the mitochondria were incubated with HEPES that permeabilizes the outer membrane, and proteins of the matrix such as mitochondrial heat shock protein 70 (mtHSP70) disappeared when mitochondria were treated with triton that disrupts both the outer and the inner membrane (Figure 3D). These results demonstrated that caspase-3 and caspase-7 activated in CSF-1-treated monocytes were located to the outer mitochondrial membrane.

Caspase-8 is another caspase activated in CSF-1-treated monocytes [13]. Its activation involves the formation of a molecular platform that associates this protease to the adaptor protein FADD in the absence of any death receptor [15,16]. Fluorescence of FAM-LETD-FMK, used as a reporter of caspase-8 activity, was detected in the cytoplasm of CSF-1-treated monocytes but did not co-localize with the mitochondria (Figure S4A). Accordingly, immunoblot analysis of mitochondria collected from CSF-1-treated monocytes showed that, while caspase-3 and -7 expression levels remained unchanged, caspase-8 disappeared from these organelles (Figure 3A). A duolink in situ proximity ligation assay used to visualize caspase-8/FADD interaction detected a signal in the cytoplasm of CSF-1-treated monocytes (Figure S4B,C), which was independent of the mitochondria (Figure S4D).

### 2.3. Caspase Activation Promotes the Generation of Cytosolic Radical Oxygen Species

**Figure 4:**
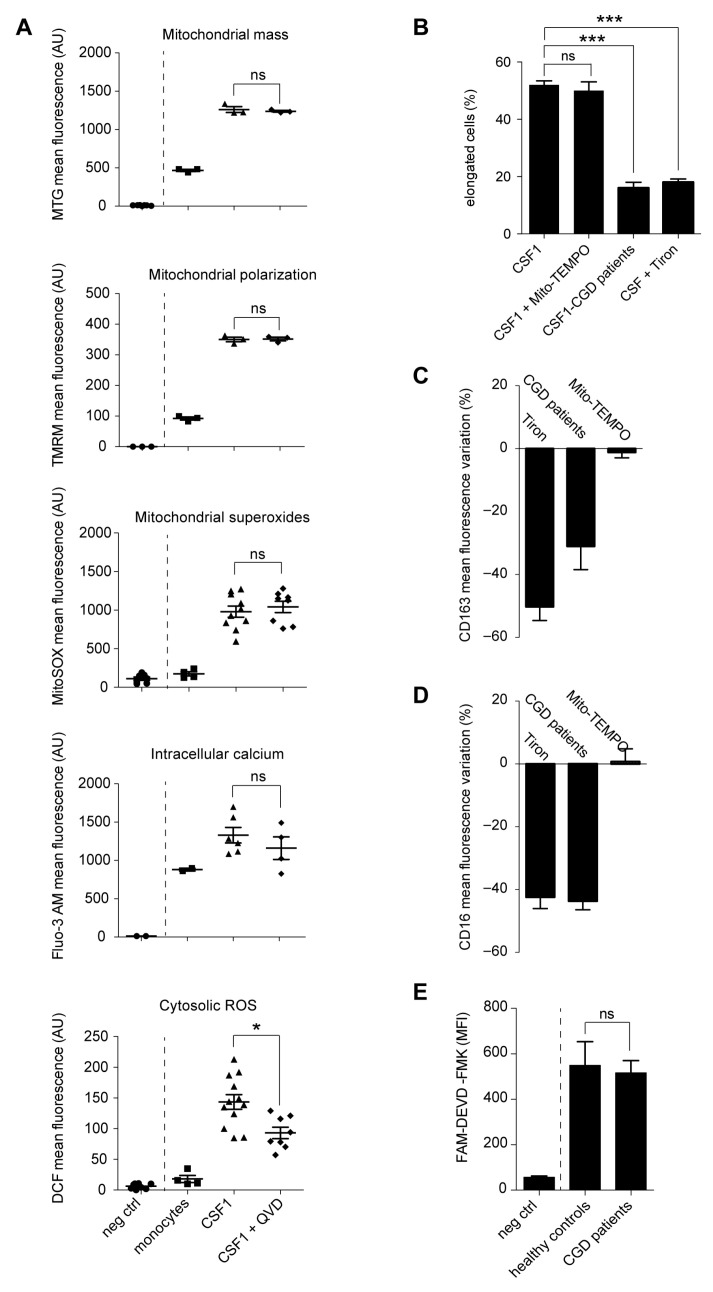
NOX2-dependent cytosolic ROS production, downstream of caspase activation. (**A**) Monocytes were untreated or treated with CSF-1 (100 ng/mL, 4 days) in the absence or presence of 50 µM Q-VD-OPh before measuring mitochondrial mass with MTG (MitoTracker Green), mitochondrial membrane potential with TMRM (tetramethylrhodamine), mitochondrial superoxides with MitoSOX, intracellular calcium with Fluo-3 AM and cytosolic ROS with DCF (2’,7’-dichlorofluorescein). The negative control (neg ctrl) corresponded to the absence of cell incubation with MTG, TMRM, MitoSOX, Fluo-3 AM or DCF. (*, *p* < 0.05; ns, non-significant; Kruskal–Wallis test with Dunn’s post test). (**B**–**D**) Monocytes from healthy donors were treated with CSF-1 (100 ng/mL, 4 days) in the absence or presence of Tiron (500 µM), or Mito-TEMPO (50 nM), and monocytes from patients with a chronic granulomatous disease (CGD, *n* = 3) were treated with CSF-1 (100 ng/mL, 4 days). (**B**) The fraction of cells with an elongated shape (elongation factor > 2.5) was measured. (***, *p* < 0.001/6; ns, non-significant; Fisher test with Bonferroni correction). Mean ± SE. (**C**,**D**) Variation in the Mean fluorescence index of cell surface CD163 and CD16 expression in healthy donor monocytes treated with CSF-1 (100 ng/mL, 4 days) and Tiron (*n* = 7) or Mito-TEMPO (*n* = 7) and in CGD monocytes treated with CSF1 alone (*n* = 3), all compared to CSF1-treated healthy monocytes (Mean ± SE). (**E**) DEVD activity in CSF-1-treated healthy donor and CGD monocytes (Mean ± SE of three measurements; Mann–Whitney test; ns, non-significant).

CSF-1 treatment induced an increase in the mass, polarization, and superanion content of mitochondria (Figure 4A). CSF-1 also increased intracellular calcium in human monocytes (Figure 4A). None of these parameters was significantly modified by co-treatment with the caspase inhibitory molecule Q-VD-OPh. In contrast, Q-VD-OPh significantly decreased the level of cytosolic reactive oxygen species (ROS) (Figure 4A). The main source of ROS in phagocytes is NADPH (nicotinamide adenine dinucleotide phosphate) oxydases, also known as NOX enzymes, which generate superoxide anion O_2_^−^ from NADPH and oxygen [26]. Inhibition of cytosolic ROS by scavenging O_2_^−^ with Tiron (4,5-Dihydroxy-1,3-benzenedisulfonic acid disodium salt) interfered with CSF-1-induced monocyte differentiation, e.g., prevented acquisition of a characteristic fibroblast-like, elongated shape (Figure 4B) and cell surface expression of CD163 and CD16 (Figure 4C,D). Conversely, a mitochondria-targeted antioxidant agent, mito-TEMPO [(2-(2,2,6,6-Tetramethylpiperidin-1-oxyl-4-ylamino)-2-oxoethyl) triphenylphospho-nium chloride monohydrate], did not demonstrate any impact on macrophage differentiation (Figure 4B–D). Finally, monocytes from patients with a chronic granulomatous disease (CGD), which are constitutively defective in NOX2 due to mutations in one of the members of the NOX2 complex [27], demonstrated a similar defect in CSF-1-induced monocyte differentiation (Figure 4B–D), even though FAM-DEVD-FMK activity was similar to that measured in healthy controls, suggesting normal activation of caspases (Figure 4E). All together, these data point to a role for NOX2-dependent generation of cytosolic ROS in CSF-1 macrophage differentiation, downstream of caspases.

### 2.4. Active Caspase-7 Promotes NOX2 Activation through p47^PHOX^ Cleavage

**Figure 5:**
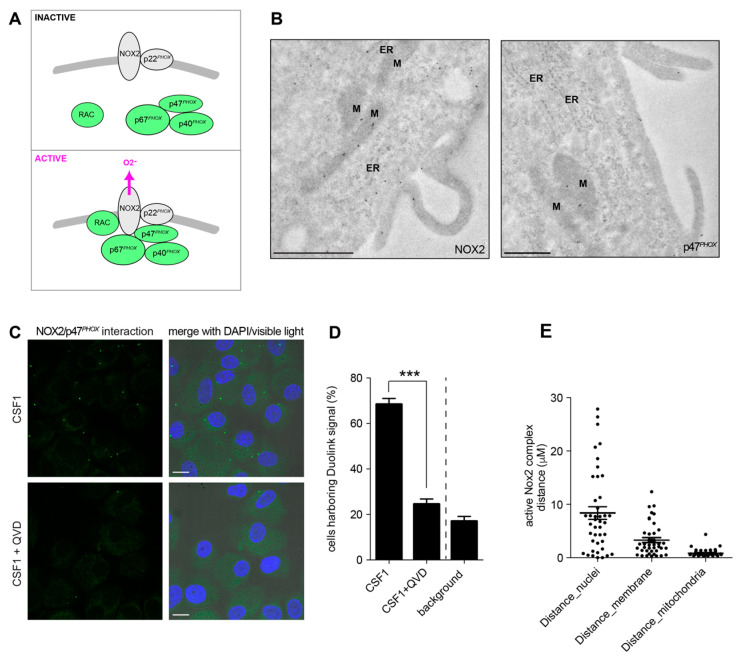
Detection of caspase dependent-active NOX2 complex in mitochondria of CSF1-treated monocytes. (**A**) Assembly of the NOX2 complex: inactive state (upper panel), active state (lower panel). (**B**) Immunogold labeling of NOX2 and p47*^PHOX^* analyzed by electron microscopy in CSF1-treated monocytes. Scale bar, 1 µm. ER, endoplasmic reticulum; M, mitochondrion. (**C**) In situ proximity ligation assay (PLA) of p47*^PHOX^* and NOX2 in CSF-1 treated monocytes (100 ng/mL, 4 days) in the absence or presence of Q-VD-OPh (50 µM). Left panel, specific signal in green; right panel, overlay with nuclear staining with DAPI in blue; Scale bar, 10 µm. (**D**) Fraction of cells harbouring a signal in PLA (***, *p* < 0.001; Fisher Exact Test). Mean ± SE. (**E**) Measurement of the distance from PLA signal to the nucleus, the membrane or the mitochondria. Circles were drawn, centered on forty-two PLA signals. The Distance_nuclei, Distance_membrane, and Distance_mitochondria correspond to the shorter radius to encounter the nucleus, the plasma membrane or the mitochondria.

**Figure 6:**
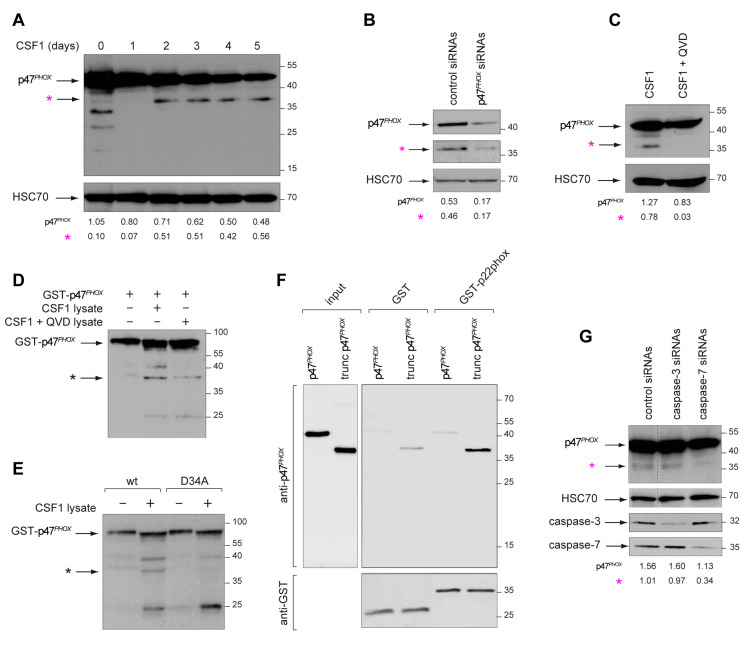
Caspase-mediated cleavage of p47*^PHOX^* generates an active NOX2 complex. (**A**) Immunoblot analysis of p47*^PHOX^* in monocytes exposed to CSF-1 for indicated times (days). HSC70, loading control; star, ≈36 kDa band. (**B**) Monocytes transfected with a pool of scrambled or p47*^PHOX^* targeting siRNAs were treated with CSF-1 (100 ng/mL) for 4 days before analysis of p47*^PHOX^* expression by immunoblotting. HSC70, loading control; star, cleavage fragment. (**C**) Immunoblot analysis of p47*^PHOX^* in cells treated for 4 days with CSF-1, with or without 50 µM Q-VD-Oph (QVD); HSC70, loading control; star, cleavage fragment. (**D**) p47*^PHOX^* protein was incubated or not with lysate (80 µg) of CSF-1 or CSF-1 + QVD-treated monocytes before immunoblot analysis with an anti-GST antibody; star, cleavage fragment. (**E**) Wild-type (wt) and D34A mutant of GST-p47*^PHOX^* protein were incubated with 80 µg of CSF-1-treated monocyte lysate before immunoblotting with an anti-GST antibody. (**F**) Full length and truncated p47*^PHOX^* were incubated with GST-p22*^PHOX^*, precipitated with glutathione-sepharose beads, and eluted with glutathione before immunoblotting with an anti-p47*^PHOX^* antibody. GST, loading control. (**G**) Monocytes transfected with a pool of scrambled or caspase targeting siRNAs were treated with CSF-1 (100 ng/mL, 4 days) before immunoblot analysis of p47*^PHOX^*, caspase-3 and caspase-7 expression. HSC70, loading control; star, cleavage fragment. Protein ladder (kDa) is indicated. Pink star: cleaved fragment from endogenous p47*^PHOX^*; black star: cleaved fragment from recombinant p47*^PHOX^*.

NOX2 activation requires interaction of four cytoplasmic proteins, p47^PHOX^, p40^PHOX^, p67^PHOX^ and RAC1 (Rac family small GTPase 1), with two membrane proteins, NOX2 and p22^PHOX^, to form a complex (Figure 5A) [28]. Electron microscopy analysis of human monocytes with immunogold labeling demonstrated that NOX2 and p47^PHOX^ could be detected at the mitochondria (Figure 5B). A proximity ligation assay detected a NOX2/p47^PHOX^ interaction in CSF-1-treated monocytes (Figure 5C–E), which was almost completely abrogated by Q-VD-OPh (Figure 5C,D), suggesting that caspase activation was required for the generation of an active NOX2 complex. Immunoblot analysis of CSF-1-treated monocytes detected a time-dependent decrease in p47^PHOX^ expression, together with the appearance of a 36-kDa peptide recognized by the p47^PHOX^ antibody (Figure 6A). The decreased detection of this 36-kDa peptide in cells treated with siRNAs targeting p47^PHOX^ further argued for a p47^PHOX^ cleavage fragment (Figure 6B). This p47^PHOX^ cleavage was blocked by co-treatment of monocytes with Q-VD-OPh (Figure 6C), suggesting the cleavage of p47^PHOX^ by caspases. Incubation of recombinant GST-p47^PHOX^ protein produced in *E. coli* with lysates of CSF-1-treated monocytes induced the cleavage of the recombinant protein, which was dramatically reduced when GST-p47^PHOX^ protein was incubated with lysates of cells co-treated with CSF-1 and Q-VD-OPh (Figure 6D). Directed mutagenesis was used to replace the aspartate residues of 9 consensus caspase-cleavage sequences identified in p47^PHOX^ sequence with alanine residues (Figure S5). Only mutation of aspartate 34 in the N-terminal PX domain of the protein could abolish p47^PHOX^ cleavage by CSF-1-treated monocyte lysate (Figure 6E and Figure S5C). We hypothesized that p47^PHOX^ cleavage at aspartate 34 could disrupt the auto-inhibitory conformation of the protein and promote its interaction with p22^PHOX^. We generated full-length and truncated p47^PHOX^ proteins in *E. coli* for pull-down experiments with GST-p22^PHOX^. Since p47^PHOX^ truncated at position 34 formed inclusion bodies in *E. coli*, we used a p47^PHOX^ truncated at position 82, which allowed showing that p47^PHOX^ truncated in its PX domain had a higher affinity for p22^PHOX^ compared to full-length p47^PHOX^ (Figure 6F). Finally, as both caspase-3 and caspase-7 are activated at the mitochondria level in CSF-1-treated monocytes, we tested which of these two enzymes could cleave p47^PHOX^. siRNA experiments showed that caspase-7 but not caspase-3 targeting siRNAs could prevent the cleavage of p47^PHOX^ in CSF-1-treated monocytes (Figure 6G). All together, these results identify a non-conventional pathway to the formation of an active NOX2 complex through caspase-7 mediated p47^PHOX^ cleavage.

### 2.5. Caspase Inhibition Alters the Migration of CSF-1 Induced Macrophages

**Figure 7:**
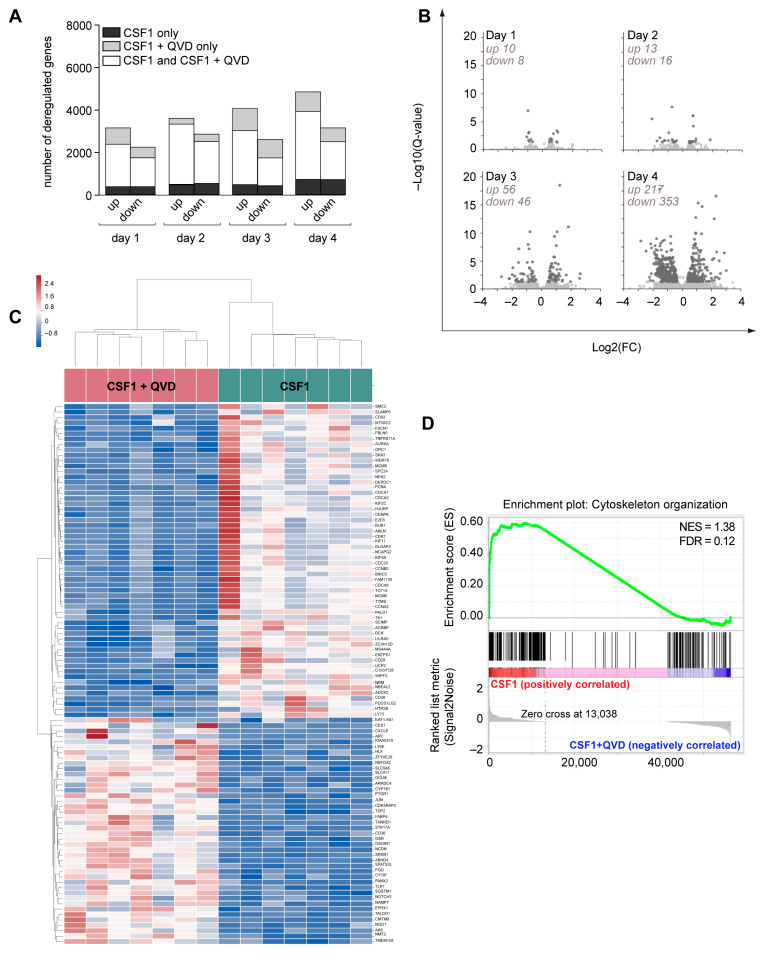
Impact of Q-VD-OPh on gene expression (mRNA) in CSF1-treated monocytes. Monocytes sorted from the peripheral blood of 7 healthy donors were treated by 100 ng/mL CSF1 in the absence or presence of 50 µM Q-VD-Oph (QVD) for 1, 2, 3 or 4 days. (**A**) Time dependent changes in the number of up- and down-regulated genes in CSF1-treated monocytes only (in black), in monocytes treated with CSF1 + QVD only (in gray), and in both situation (in white). (**B**) Volcano plots of differentially expressed genes in CSF1 + QVD compared to CSF1-treated monocytes (dark gray, qvalue < 0.05, fold change > 1.3). (**C**) Heatmap of the expression of the top-100 deregulated genes in monocytes treated with CSF1 alone (right) and CSF1 + QVD (left). (**D**) Gene Set Enrichment Analysis (GSEA) of CSF1 + QVD-treated monocytes compared to CSF1-treated monocytes. NES, normalized enrichment score; FDR, false discovery rate.

To gain insights into the role of caspases in CSF-1-induced macrophages, we serially measured the level of eight cytokines in the supernatant of CSF-1-treated monocytes and failed to detect any significant impact of Q-VD-OPh on cytokine production (Figure S6). We also performed sequential RNA sequencing of sorted monocytes treated ex vivo with CSF-1 in the absence or presence of Q-VD-OPh for up to 4 days. These analyses detected CSF-1-induced time-dependent changes in gene expression in the presence and absence of Q-VD-OPh (Figure 7A). Principal Component Analysis (PCA) of gene expressed in the absence and presence of Q-VD-OPh identified an effect of monocyte donor on PC1 and an effect of treatment on PC2 (Figure S7A). qPCR analysis of samples used for RNA sequencing validated the observed changes in 9 out of 9 selected genes (Figure S7B). Comparison of CSF-1 and CSF-1 + Q-VD-OPh-treated monocytes demonstrated a time-dependent increase in the number of differentially expressed genes, reaching 353 down-regulated and 217 up-regulated genes at day 4 (Figure 7B). The heatmap of differentially expressed genes at day 4 demonstrates the significant impact of Q-VD-OPh on CSF-1-treated monocytes (Figure 7C). Gene ontology analyses of differentially expressed genes suggested a link with cytoskeleton organization, which was also the most enriched term identified by gene set enrichment (Figure 7D). These results suggested that functions of macrophages recruited upon CSF-1 stimulation of monocytes could be modulated by caspase inhibition.

**Figure 8:**
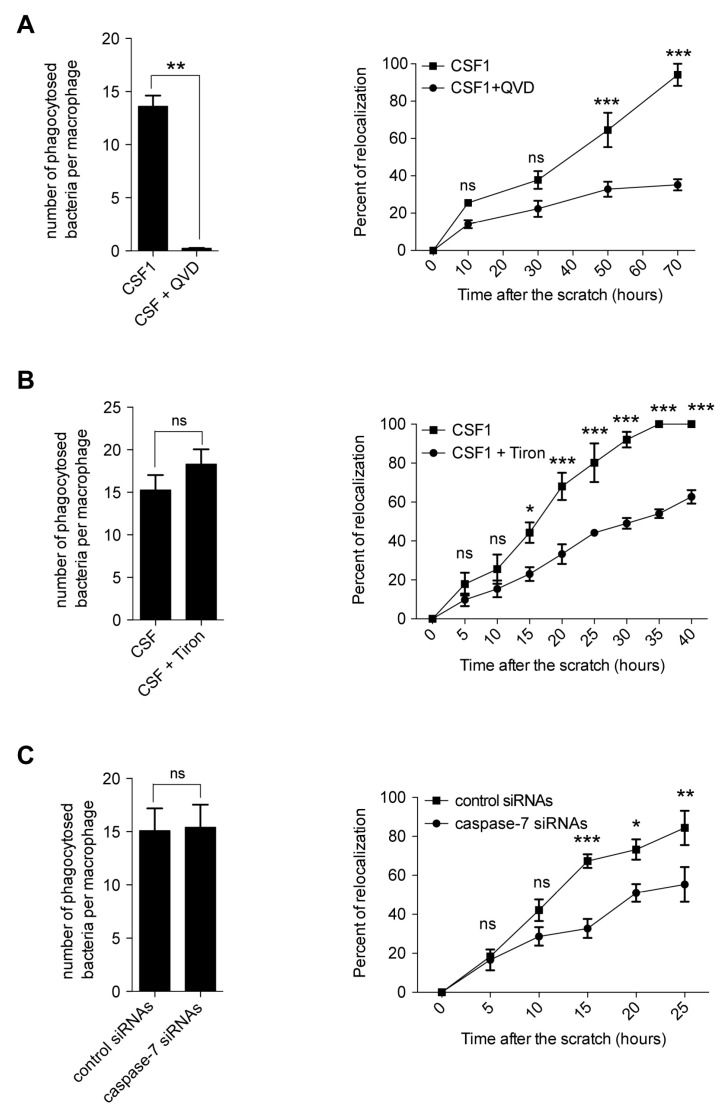
Caspase inhibition alters the migration of CSF1-induced macrophages. Bacterial uptake was measured by the gentamicin protection assay (left panels) and cell migration was studied using a wound-healing assay (right panels) in monocytes treated for 4 days with CSF-1, with or without 50 µM Q-VD-OPh (**A**), 500 µM Tiron (**B**), or a pool of scrambled or caspase-7 targeting siRNAs (**C**). Mann–Whitney test was used to compare the number of phagocytosed bacteria, and two-way ANOVA with Bonferroni post-test was used for the wound-healing assay (*, *p* < 0.05; **, *p* < 0.01; ***, *p* < 0.001; ns, non-significant). Mean ± SE.

As cytoskeleton was among the most enriched gene sets in CSF-1 + Q-VD-OPh-treated monocytes at day 4, we focused on the potential impact of caspase inhibition on macrophage phagocytosis and migration. Q-VD-OPh could prevent the ability of CSF-1-induced macrophages to engulf *E. coli*. However, this function was not altered in the presence of Tiron, the cytosolic ROS scavenger, and when caspase-7 was down-regulated with a pool of siRNAs in these cells (Figure 8A–C, left panels). We also performed a wound-healing assay, showing that caspase inhibition with Q-VD-OPh decreased the migration capabilities of CSF-1-induced macrophages (Figure 8A, right panel). This assay showed that migration of CSF-1-treated monocytes was also reduced by scavenging O_2_^−^ with Tiron (Figure 8B, right panel), by inhibiting NOX with NS1 [29] and by a pool of siRNAs mediating caspase-7 down-regulation (Figure 8C, right panel). Collectively, our results argue for a role of caspase-7, activated at the surface of the mitochondria in NOX2 activation and the generation of cytosolic ROS that contribute to the motility of macrophages.

### 2.6. Caspase Inhibition in Monocytes/Macrophages Prevents Bleomycin-Induced Lung Fibrosis

**Figure 9:**
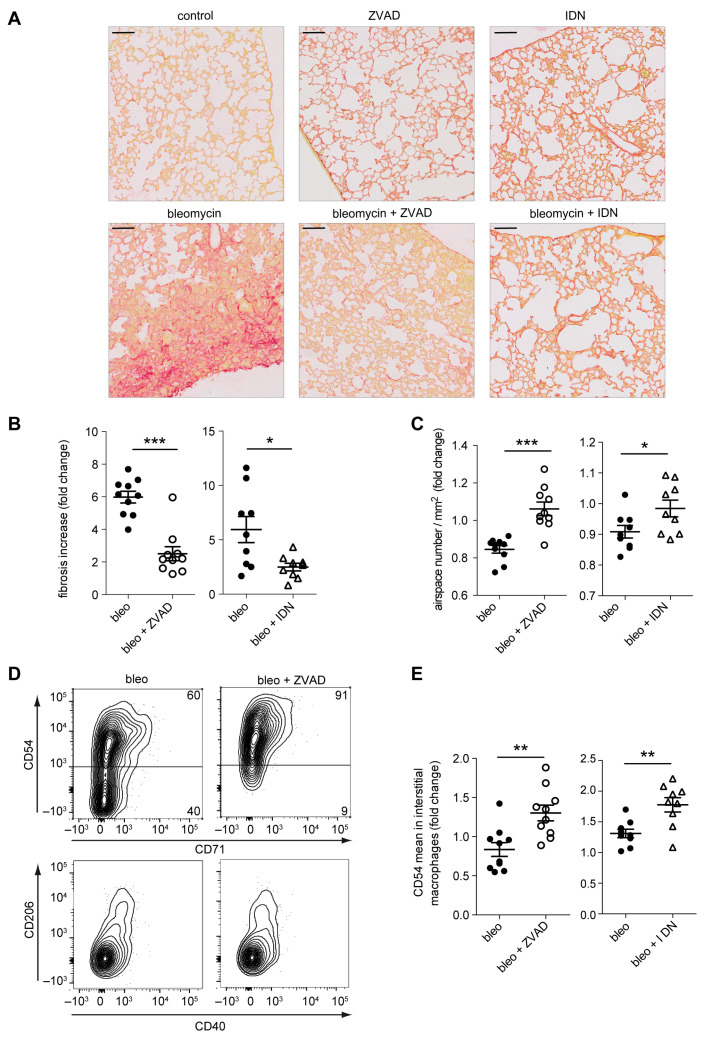
Emricasan prevents bleomycin-induced lung fibrosis. (**A**) Representative histological pictures of Sirius Red-stained lung tissue sections from untreated mice (control) and mice injected intraperitoneally with bleomycin sulphate (0.1 mg/g body weight) once a week for 3 weeks, without (bleomycin) or with Z-VAD-FMK (bleomycin + ZVAD, 1 µg/g body weight intra-peritoneally every day for three weeks) or Emricasan (bleomycin + IDN, 18 µg/g body weight subcutaneously twice a day during three weeks) and mice treated with Z-VAD-FMK (ZVAD) or Emricasan (IDN) alone. Lungs were fixed in FineFix and paraffin embedded before staining collagen fibers with Sirius Red. Scale bar, 100 µm. (**B**) Quantification of Sirius Red labeling intensity. Results are expressed as fold change in Sirius Red staining in treated compared to control mice (bleomycin was compared to untreated, bleomycin + ZVAD to ZVAD alone, bleomycin + IDN to IDN alone). Each dot or square is an individual mouse. ***, *p* < 0.001; *, *p* < 0.05; Mann–Whitney test. Mean ± SE. (**C**) Quantification of airspace number/mm^2^ of parenchymal tissue. Results expressed as fold change in treated compared to control mice as in B. ***, *p* < 0.001; *, *p* < 0.05; Mann–Whitney test. Mean ± SE. (**D**) Flow cytometry analysis of CD54, CD71, CD206 and CD40 at the surface of interstitial macrophages from bleomycin- and bleomycin + ZVAD-treated mice. Representative scatter plots are shown. (**E**) CD54 mean fluorescence intensity at the surface of lung interstitial macrophages expressed in fold change (**, *p* < 0.01; Mann–Whitney test). Mean ± SE.

**Figure 10:**
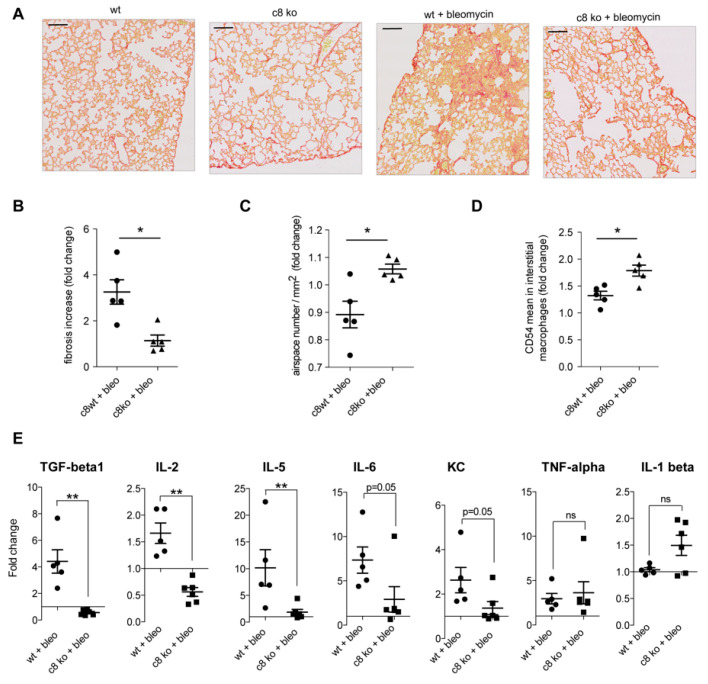
Caspase-8 deletion in granulo-monocytes prevents bleomycin-induced lung fibrosis. (**A**) Representative histological pictures of Sirius Red-stained lung tissue sections from wild-type (wt) and LysM-Cre; *Caspase-8^flox/flox^* (c8 ko) mice either left untreated (left panels) or injected intraperitoneally with bleomycin sulphate (0.1 mg/g body weight) once a week for 3 weeks (right panels). As in Figure 9A, lungs were fixed in FineFix and paraffin embedded before staining collagen fibers with Sirius Red. Scale bar, 100 µm. (**B**) Quantification of Sirius Red labeling intensity. Results are expressed as fold change in Sirius Red staining in treated compared to untreated control mice (wt + bleomycin was compared to wt, c8 ko + bleomycin was compared to c8 ko) *, *p* < 0.05; Mann–Whitney test. Mean ± SE. (**C**) Quantification of airspace number/mm^2^ of parenchymal tissue. Results are expressed as fold change in treated compared to untreated wild-type mice, as in panel B; *, *p* < 0.05; Mann–Whitney test. Mean ± SE. (**D**) Flow cytometry analysis of CD54 mean fluorescence intensity at the surface of lung interstitial macrophages expressed in fold change (*, *p* < 0.05; ns, non-significant; Mann–Whitney test). Mean ± SE. (**E**) Cytokines were measured in broncho-alveolar lavage fluid collected from bleomycin-treated wild-type (wt) and LysM-Cre/Casp8^fl/fl^ (c8 ko) mice treated with bleomycin. Results are expressed as fold-changes compared to untreated mice (**, *p* < 0.01; ns, non-significant; Mann–Whitney test). Mean ± SE.

The impact of the caspase/NOX2 axis on the phenotype and function of CSF1-induced macrophages suggested that their inhibition could have a therapeutic effect. To explore this hypothesis, we choose bleomycin-induced lung fibrosis in mice as “M2”-polarized macrophages [30] and NOX2-mediated production of ROS [31] play an important role in the pathogenesis of lung fibrosis. Mice injected intraperitoneally by bleomycin sulphate once a week develop a lung fibrosis that, after three weeks, can be visualized by Sirius Red staining of collagen fibers (Figure 9A,B) and airspace (Figure 9C). Lung fibrosis intensity was less important in mice treated by the pan-caspase inhibitor, Z-VAD-FMK (carbobenzoxy-valyl-alanyl-aspartyl-[O-methyl]- fluoromethylketone) (Figure 9A–C). Subcutaneous injection of Emricasan (IDN-6556), a pan-caspase inhibitor currently developed in clinics, demonstrated the same ability to prevent lung fibrosis development (Figure 9A–C). This was associated with an increase expression of CD54 at the surface of interstitial macrophages (Figure 9D,E). Finally, we observed that lung fibrosis was less important in mice with monocyte-restricted deletion of caspase-8 (LysM-Cre; Casp8^fl/fl^) (Figure 10A–C), which was associated with an increased expression of CD54 on interstitial macrophages (Figure 10D) and a decreased quantity of TGF-beta1 (Transforming growth factor beta 1), as well as several inflammatory cytokines in the broncho–alveolar lavage fluids (Figure 10E).

## 3. Discussion

While caspases are best characterized for their conserved roles in the orchestrated program of apoptotic cell death, they have also demonstrated non-apoptotic functions, e.g., CED-3, the founding member of the caspase family identified in *C. elegans*, is involved in processes ranging from neuronal regeneration to aging [32]. In the hematopoietic tissue, caspases exert non-apoptotic functions in specific cell lineages [33], i.e., some of these enzymes regulate the timely controlled removal of organelles during erythroid differentiation [34] and promote platelet biogenesis in situations of acute need [35]. These non-apoptotic functions require protective mechanisms to prevent unwanted cell death. In human erythroid cells, a complex interplay between caspase-3 activation, the key transcription factor GATA-1 and the chaperone protein HSP70, protects GATA-1 from being cleaved [36,37]. Here, we show the spatial restriction of active caspase-3 and caspase-7 in CSF-1-treated monocytes. A spatial restriction of active caspases has been described in Drosophila, i.e., the basal side of the plasma membrane is a non-apoptotic environment for caspase function in so-called apoptosis-induced proliferation [38], and individualization of spermatids requires caspase activation at the surface of the mitochondria [39,40]. Caspase-3 and -7 activities at the mitochondria level depicted in CSF-1-treated human monocytes is another declension of the spatial control of active caspases in cells that are not dedicated to death.

Caspases are synthesized as zymogens and exhibit a variety of activation mechanisms. In dying cells, initiator caspases are monomeric proenzymes that become activated upon dimerization within oligomeric signaling platforms in which they are recruited, [41] while effector caspases are pre-formed dimers of proenzymes that become catalytically competent on proteolytic cleavage by initiator caspases [42,43]. Additional modes of caspase regulation have been described [44,45,46], with recent analyses demonstrating that zymogens as well as processed forms of caspase-3 and -7 could adopt either active or inactive conformations [47], indicating a variety of strategies to modulate their activities [48,49]. In situ enzyme trapping experiments suggest that, in CSF-1-treated monocytes, the catalytic activity of caspase-3 and caspase-7 is bound by the full-length pro-enzyme and, with respect to caspase-7, an alternatively processed form. Broad spectrum caspase inhibitors Q-VD-OPh and Emricasan, which inhibit apoptosis-related caspase cleavage, also impair the cleavage of caspases at their specific differentiation cleavage site.

Caspase-8, which is typically processed following engagement of a death receptor [50,51], can also be catalytically activated in the absence of cleavage, e.g., in T cells expanding upon T cell receptor engagement [52]. This enzyme has demonstrated both pro-apoptotic and pro-survival functions, which may be promoted in distinct protein complexes [53,54]. In CSF-1-treated monocytes, caspase-8 is recruited in a cytosolic platform that includes FADD and FLIP_L_ [16], which is reminiscent of the pro-inflammatory FADDosome identified in cells exposed to TRAIL (TNF-related apoptosis inducing ligand) [55]. In *C. elegans*, non-apoptotic functions of CED3 also require the formation of a multiprotein complex that is not observed in the apoptotic setting [56]. The FADD/caspase-8 complex detected in CSF-1-treated monocytes localizes in the cytoplasm, not in the mitochondria vicinity, indicating that several mechanisms operate to prevent activated caspases from killing the cells and suggesting that the diverse active caspases may have distinct non-apoptotic roles.

The NADPH oxidase NOX2 has been predominantly involved in the formation of ROS in monocytes [26]. We show that CSF-1-mediated differentiation of monocytes collected from patients with CGD, an inherited disorder of NOX2 in which patients suffer from life-threatening infections [57], is altered. In the cytosol, the NOX2 complex component p47*^PHOX^* is auto-inhibited by interaction of its two SH3 (src Homology 3) domains with its C-terminal region [58]. Typically, phosphorylation of p47*^PHOX^* disrupts inhibitory interactions within the protein to promote the assembly of active NOX2 complex [59]. Here, we depict an alternative mode of NOX2 activation through truncation of p47*^PHOX^* at the PX domain, which increases its affinity for p22*^PHOX^*. Similarly, the Drosophila NADPH-oxidase Duox was shown to be activated by the caspase Dronc in a non-apoptotic context [38].

Gene expression analysis was used to further identify the effects of caspase inhibition on CSF-1-treated monocytes. Gene ontology of modified gene sets identified cytoskeleton as the most enriched terms in the presence of Q-VD-OPh. Accordingly, this compound could decrease the phagocytic and migration properties of CSF-1-treated monocytes. To get further insights on the specific effect of caspase-7, we decreased its expression and inhibited superoxide anion (O_2_^−^) whose generation involves the active enzyme. The decreased migration observed in both situations was in accordance with the function of NOX2 in the migration of macrophages in response to CSF-1 stimulation [60]. The lack of impact of caspase-7 down-regulation and superanion oxide inhibition on phagocytosis, which is otherwise inhibited by Q-VD-OPh, suggests either the regulation of phagocytosis by another active caspase, or a non-specific, caspase-independent effect of the inhibitory compound.

The functional plasticity of macrophages had led to therapeutic manipulation [61]. The ability of caspase deletion or inhibition to prevent the development of bleomycin-induced lung fibrosis is associated with changes in the cell surface markers of lung-infiltrating macrophages and the level of cytokines detected in the broncho-alveolar lavage, suggesting modification in the polarization of these cells. Pan-caspase inhibition thus appears as an alternative strategy to monoclonal antibodies and small molecule inhibitors of CSF1 and CSF1R [10,62], STAT3 inhibitory molecules [63], DNA vaccine against the “M2”-associated asparaginyl endopeptidase legumain [64] and other strategies [65] to modulate the polarization of macrophages recruited in damaged tissues.

## 4. Materials and Methods

### 4.1. Study Design

Macrophage polarization plays an important role in the pathogenesis of diverse human diseases as cancer, leading us to explore if caspase inhibition would affect macrophage polarization. To explore the role of caspases in CSF1 differentiation, we used human monocytes sorted from buffy coats or from blood of NOX2-deficient patients treated by cytokines, and we generated monocyte-restricted caspase-8 knockout and caspase-3/caspases-7 double knockout mice, which were treated with bleomycin to induce pulmonary fibrosis.

To study the impact of Z-VAD-FMK on bleomycin-induced lung fibrosis, we first realized an experiment with 5 mice by group (standart procedure in the laboratory). In presence of Z-VAD-FMK, we observed a decrease of pulmonary fibrosis, which was not statistically significant; therefore, we realized a second similar experiment (*n* = 5) in order to increase the number of mice by group. The analysis of the pool of the 2 experiments showed that Z-VAD-FMK significantly decreased the pulmonary fibrosis (Mann Whitney test, *p* = 0.0003, two-tailed). Based on these results, we estimated the sample size in order to have sufficient statistical power (80%) to highlight the potential efficacy of IDN-6556 and caspase-8 invalidation with a Wilcoxon Mann–Whitney test, assuming that IDN-6556 and caspase-8 invalidation will have at least the same efficacy than Z-VAD-FMK. The sample size calculation using simulations based on normal distribution assumption of the distribution in each group indicated that 5 mice were sufficient (corresponding to 84.5% of statistical power). As sensibility analysis, to avoid making a normal assumption, we realized the sample size calculation using bootstrap procedure which indicated that 6 mice were sufficient (corresponding to 88.9% of statistical power). Therefore, the future experiments were performed with at least 5 or 6 mice.

The results from the in vitro experiments were at least from three independent experiments.

No data were excluded from the analyses and all attempts at replication were successful. For the mice experiments, the animals were allocated to groups randomly and the investigators were blinded during Sirius Red staining analysis.

### 4.2. Chemical Reagents and Antibodies

CSF1, GM-CSF, IL-4, Q-VD-OPh and Z-VAD-FMK were obtained from R&D Systems (Minneapolis, MN, USA). Ac-YVAD-cmk from InvivoGen (San Diego, CA, USA), staurosporine and cleaved caspase-3 blocking peptide (#1050) from Cell Signaling (Danvers, MA, USA), IDN-6556 from MedChemtronica (Stockholm, Sweden), Tiron, TEMPO and Mito-TEMPO from Santa Cruz (Dallas, TX, USA). We used antibodies to calreticulin (#ab22683, Abcam, Cambridge, MA, USA), active caspase-3 (#9664, Cell Signaling), caspase-3 (#7148, Santa Cruz), caspase-3 (#9662, Cell Signaling), caspase-7 (#9492, Cell Signaling), caspase-7 (#sc-81654, Santa Cruz), caspase-8 (#9746, Cell Signaling), caspase-8 (#AF705, R&D Systems), DIABLO (#2409, ProSci, Poway, CA, USA), FADD (#2782, Cell Signaling), GST (#sc-138, Santa Cruz), γ-H2AX (#05-636, Merck Millipore, Billerica, MA, USA), HSC70 (#ADI-SPA-816, Enzo Life Sciences, Farmingdale, NY, USA), mtHSP70 (#MA3-028, Thermo Fisher Scientific, Waltham, MA, USA), LC3 (#M152-3, MBL International, Woburn, MA, USA), NOX2 (#sc-130543, Santa Cruz), p47*^PHOX^* (#4312, Cell Signaling), TOM20 (#sc-11415, Santa Cruz).

### 4.3. Cell Culture

Peripheral blood samples were collected from healthy donors by the Etablissement Français du Sang without further information, and from three patients with a chronic granulomatous disease with the approval of Paris-Descartes University ethical committee and their written informed consent. CD14^+^ monocytes were sorted using microbeads according to the manufacturer’s instructions (Miltenyi Biotec, Somerville, MA, USA), cultured in RPMI 1640 with glutamine (Thermo Fisher Scientific) supplemented with 10% fetal bovine serum and exposed to 100 ng/mL CSF-1 or GM-CSF to generate their differentiation, in the absence or presence of 50 µM Q-VD-OPh, 50 µM Ac-YVAD-cmk, 500 µM Tiron, or 50 nM Mito-TEMPO. Mitochondrial mass, mitochondrial superoxides, intracellular calcium and cytosolic ROS were measured by incubating the cells with 100 nM MitoTracker green (30 min at 37 °C) or 2.5 µM MitoSOX Red Mitochondrial Superoxide Indicator (10 min at 37 °C) or 2 µM Fluo-3-AM for (30 min at 37 °C) or 0.5 µM H2DCFDA (30 min at 37 °C) (all by Thermo Fisher Scientific), respectively, before flow analysis of fluorescence intensity. When indicated, monocytes were transfected with Human Monocyte Nucleofector kit (Lonza, Atlanta, GA, USA). Briefly, 5 × 10^6^ cells were suspended into 100 µL of nucleofector solution with 200 pmol of ON-TARGETplus p47*^PHOX^* SMARTpool siRNA (#L-180696-01, GE Dharmacon, Lafayette, CO, USA), ON-TARGETplus caspase-3 SMARTpool siRNA (#L-004307-00, GE Dharmacon), ON-TARGETplus caspase-7 SMARTpool siRNA (#L-004407-00, GE Dharmacon) or ON-TARGETplus Non-targeting pool before nucleofection with Nucleofector II (Lonza), incubated overnight with 5 mL of pre-warmed complete RPMI medium (Thermo Fisher Scientific), and exposed to CSF-1 the following day.

### 4.4. Flow Cytometry Analysis of Cell Phenotype

Cells were washed with ice-cold PBS, incubated with Fc block (Human TruStain FcX, Biolegend, London, UK, 1/20) for 15 min, incubated with antibodies for 20 min at 4 °C and washed before analysis using a BD LSRFortessa X-20. Antibodies were AlexaFluor700-CD80 (#561133, BD, Franklin Lakes, NJ, USA), PE/Cy7-CD86 (#561128, BD), APC/Alexa750-CD71 (#A89313, Beckman coulter, Brea, CA, USA), ECD-CD62L (#IM2713U, Beckman coulter), Krome orange-CD14 (#B01175, Beckman coulter), Pacific Blue-CD16 (#A82792, Beckman coulter), APC-CD1a (#IM3645, Beckman coulter), PE-CD163 (#556018, BD) and FITC-CD115 (#FAB329F, R&D Systems). The data were analyzed with FlowJo software v. 10.0.00003.

### 4.5. Cell Morphology

Phase contrast images were captured with a Nikon Eclipse TE300 microscope before manually tracing the long and short axes of each cell (long axis, longest length of the cell; short axis, length across the nucleus in a direction perpendicular to the long axis) to measure the elongation factor as the ratio of these axes [66].

### 4.6. Immunofluorescence Microscopy

Cells were washed in PBS, fixed with 4% formaldehyde in PBS for 20 min, washed with PBS, post-fixed and permeabilized with cold 70% ethanol for 20 min, washed with PBS, blocked with 8% bovine serum albumin (BSA) in PBS for 1 h, incubated with the first antibody (active caspase-3, 1/250; γ-H2AX, 1/500; calreticulin, 1/250; LC3, 1/250) in 1% BSA in PBS for 2 h, washed and incubated with a secondary antibody conjugated with Alexa Fluor 488 or 555 for 1 h, washed and mounted by using Vectashield mounting medium with DAPI (Vector Laboratories, Burlingame, CA, USA). Mitotracker orange (#M7510, Thermo Fisher Scientific, 100 nM) was incubated 30 min with living cells at 37 °C. FAM-DEVD-FMK and FAM-LETD-FMK detection kit FLICA (Bio-Rad, Marnes-la-Coquette, France) was used according to the manufacturer’s instructions. Confocal images were acquired on a Leica SPE Confocal system, sequential acquisition using 63X/1.3 oil immersion. For 3D pictures, we used Bitplane Imaris software v. 7.7.2. Lines intensity profiles were realized using the LAS AF software v. 2.7.3.9723. Fluorescence quantification and co-localization quantification were calculated using ImageJ 2.0.0-rc-69/1.52p.

### 4.7. In Situ Proximity Ligation Assay

Cells were fixed with 4% formaldehyde in PBS for 20 min, permeabilized with cold (−20 °C) 70% ethanol for 20 min, blocked with 8% BSA in PBS for 1 h, incubated with the first antibody (caspase-8, 1/50 dilution; FADD, 1/100 dilution; NOX2, 1/250 dilution; p47*^PHOX^*, 1/250 dilution) in 1% BSA in PBS for 1.5 h, then with PLUS and MINUS PLA probes (Olink Bioscience, Uppsala, Sweden) for 1 h at 37 °C, then with ligation mix (Olink Bioscience) for 30 min at 37 °C, then with amplification mix (Olink Bioscience) for 100 min at 37 °C before being mounted using Duolink In Situ Mounting Medium with DAPI (Olink Bioscience). Confocal images were acquired on a Leica SPE Confocal system, sequential acquisition using 63X/1.3 oil immersion. 3D pictures were realized with the Bitplane Imaris software v. 7.7.2.

### 4.8. Electron Microscopy

Cells fixed for 1 h at 4 °C in 1.6% glutaraldehyde in Sörensen’s phosphate buffer pH 7.2 were dehydrated in methanol and embedded in Lowicryl K4M at −20 °C under UV using a Leica EM AFS2/FSP automatic reagent handling apparatus (Leica Microsystems). Thin-sections were incubated with a primary antibody (anti-active caspase-3, #9664, Cell Signaling; anti- p47*^PHOX^* antibody, #4312, Cell Signaling; anti-NOX2, #sc-130543, Santa Cruz), for 1 h at room temperature, then with a gold-conjugated goat anti-rabbit antibody (BBInternational, Cardiff, UK), both diluted 1/25 in PBS. After staining with 4% aqueous uranyl acetate, grids were observed under a Tecnai 12 electron microscope (FEI) and digital images taken with a SIS MegaviewIII charge-coupled device camera (Olympus).

### 4.9. Caspase Activity

FAM-DEVD-FMK labels activated caspase 3 and 7 in living cells while FAM-LETD-FMK labels activated caspase-8. These probes enter the cells and become covalently coupled to the active enzymes while unbound probe is washed away. The green, fluorescent signal (measured by flow cytometry) indicates the caspase enzyme activity present in the cell at the time the reagent was added. Mitochondrial activity was measured in cells labeled with FAM-DEVD-FMK and 150 nM TMRM (Thermo Fisher Scientific) for 30 min at 37 °C before sorting mitochondria using TOM22 microbeads (Miltenyi Biotec) to measure DEVD activity.

### 4.10. Immunoblotting

Cells were washed twice in PBS and lysed at 4 °C (1% SDS and 10 mM Tris-HCl, pH 7.4) supplemented with protease and phosphatase inhibitors. Viscosity was reduced by sonication and equal amounts of proteins were boiled for 5 min in Tris-glycine-SDS sample buffer (Thermo Fisher Scientific), separated by Tris-glycine polyacrylamide gels, and electroblotted onto nitrocellulose membranes (Bio-Rad). Membranes saturated with milk were incubated overnight at 4 °C with primary antibodies, then for 45 min with peroxydase-conjugated anti-IgG secondary antibodies (Santa Cruz). Signals were revealed by autoradiography using the Enhanced Chemiluminescence detection kit (Thermo Fisher Scientific). Mitochondria were sorted using the Qproteome Mitochondria isolation kit (QIAGEN, Germantown, MD, USA) or recovered by centrifugation at 10,000 g from cells lysed with a dounce homogenizer after removal of nuclei and cell debris (2000 g), then suspended in 20 mM Hepes or in isolation buffer (10 mM Hepes pH 7.4 NaOH, 210 mM mannitol, 70 mM sucrose, 1 mM EDTA) without or with 0.2% Triton X-100. Proteinase K (5 ug) was added when required (30 min on ice before adding PMSF). Samples were precipitated with 12% TCA before immunoblotting.

### 4.11. In Situ Caspase Trapping

Cells were incubated with 20 µM biotin-VAD-fmk or biotin-DEVD-fmk (MP Biomedicals, Solon, OH, USA) for 10 min at 37 °C before lysis and capture of biotinylated proteins on streptavidin beads (Thermo Fisher Scientific), which were suspended in Laemmli buffer with beta-mercaptoethanol before immunoblotting.

### 4.12. Phagocytosis

We used a gentamicin protection assay. CSF-1-treated monocytes (0.25 million) were infected with ampicillin resistant *E. coli* K12 (MOI = 300) (Thermo Fisher Scientific) for 20 min in RPMI 1640 (Thermo Fisher Scientific) supplemented with 10% fetal bovine serum. Then, the cells were washed three times, incubated with RPMI 1640 supplemented with 10% fetal bovine serum and gentamicin (50 μg/mL) (Thermo Fisher Scientific) and lysed in PBS with 0.1% Triton X-100. Bacteria that penetrated the cell were released by Triton treatment and plated on LB plates containing ampicillin (100 μg/mL). Colonies were counted on the next day, allowing to calculate the number of bacteria ingested macrophages.

### 4.13. Wound-Healing Assay

Human or murine macrophages were treated with CSF-1, seeded at high density on day 4, scraped at day 5 with a 1–10-μL tip and washed before measuring recolonization by video microscopy during a 60 to 70-h period.

### 4.14. Cytokine Profile in Human Macrophages Supernatants

IL-1 beta, IL-4, IL-6, IL-8, IL-10, IL-12, IFN-gamma and TNF-alpha concentrations were measured using the human Pro-Inflammatory Combo 1 U-Plex (MSD, Rockville, MD, USA) in cell culture supernatant collected every day. Chemiluminescence signal was measured on a Sector Imager 2400 (MSD).

### 4.15. p47^PHOX^ Proteolytic Cleavage

The Q5 Site-Directed Mutagenesis kit (New England BioLabs, Ipswich, MA, USA) was used to introduce point mutations in GST-p47*^PHOX^* construct [67] or delete the first 82 amino acids of p47*^PHOX^*. Primers used for mutagenesis are listed in Table S1. PCR were carried out for 25 cycles. Recombinant proteins were generated by subcloning GST-p47*^PHOX^* in pGEX-6P-3 before expression in *E. coli* (BL21) (Merck Millipore), which were inoculated overnight in 100 mL of LB medium containing 100 µg/mL ampicillin at 37 °C. The overnight culture diluted 10-fold was allowed to grow until the optical density at 600 nm reached 0.9. Expression of p47*^PHOX^* was induced with 0.1 mM isopropyl B-D-thiogalactoside (SIGMA-ALDRICH) for 3 h at 37 °C. Recombinant proteins were purified with glutathione (GSH) sepharose beads (SIGMA-ALDRICH, Saint-Quentin Fallavier, France). In vitro caspase cleavage assay was performed by incubating recombinant wild-type and mutated p47*^PHOX^* (300 ng) in caspase buffer (50 mM Hepes pH 7.2, 50 mM NaCl, 0.1% CHAPS, 10 mM EDTA, 5% glycerol, 10 mM DTT) with 80 µg of CSF-1-treated monocytes fresh lysate, for 3 h at 37 °C before immunoblotting. GST pull-down assay was done by incubating 214 nM recombinant wild-type p47*^PHOX^* and p47*^PHOX^* (83–390) with 286 nM of GST or GST-p22*^PHOX^* (132–195) for 30 min at 4 °C in PBS, pH 7.4, 0.1% Triton X-100. Glutathione sepharose beads were added for 1 h at 4 °C before elution in Tris 100 mM, pH 7.4, 200 mM NaCl, 10 mM glutathione, and immunoblot analysis.

### 4.16. RNA Sequencing

RNA extracted using the RNeasy mini kit (QIAGEN) was processed using SureSelect Automated Strand Specific RNA Library Preparation Kit and sequenced on Illumina Novaseq 6000 in paired-end 100bp mode in order to reach at least 40 million reads per sample. Raw reads cleaning was done using Trimmomatic (v0.32) with the following parameters: ILLUMINACLIP:2:30:10 LEADING = 3 TRAILING = 3 SLIDINGWINDOW:8:20 MINLEN:30. Cleaned reads were pseudo-mapped to genome with Salmon (v0.9.2) and wasabi (v0.2) with the following parameters: --numBootstraps 100 --gcBias --seqBias --libType A. The differential analysis was performed with Sleuth (v0.29), in gene aggregation mode on Gencode HG38, with qvalue below 0.05 and fold change above 1.3. Graphs and clustered heatmap were performed with euclidian distance and ward criterion under in-house python (v3.5.2) scripts. RNA sequencing data are in the ArrayExpress database at EMBL-EBI (www.ebi.ac.uk/arrayexpress) under accession number E-MTAB-5752 (Username: Reviewer_E-MTAB-5752; Password: y9PIG6yd). Gene Set Enrichment Analysis (GSEA) was performed with the java version gsea2.0 of the Broad Institute installed on a local platform and the Molecular Signatures Database v6.2 as reference. Primers used to validate gene expression changes by qRT-PCR are listed in Table S2.

### 4.17. Animal Models

C57/BL6 female mice (8 weeks-old) were purchased from Charles River Laboratories (L’arbresle, France). *Caspase-3^flox/flox^* mice were generated using ES clone HEPDO7164G05 (C57BL/6N) from the International Mouse Phenotyping Consortium (IMPC). The neomycin selection cassette was removed using FLPe deleter mice (PMID: 10835623). *Caspase-7^flox/flox^* mice were backcrossed to C57BL/6J for 10 generations, as described (PMID: 22195746). *Caspase-3/-7^flox/flox^* LysMCre^Tg/+^ mice were generated using LysMCre transgenic mice (PMID: 10621974). *Caspase-8^flox/flox^* mice were kindly provided by Hedrick’s laboratory (UCSD) (PMID:16148088) and crossed with LysMCre transgenic mice (PMID: 10621974). Animal genotyping was done by PCR using primers indicated in Table S3, and by immunoblotting with antibodies to caspase-3 (#9662, Cell Signaling), caspase-7 (#sc-81654, Santa Cruz) and caspase-8 (#AF705, R&D Systems).

### 4.18. Murine Macrophage Generation and Analysis

Bone marrow cells were flushed from femurs and tibias, centrifuged, then exposed to geys buffer for 5 min on ice, incubated with Fc block (Murine TruStain FcX, Biolegend, 1/50 dilution) for 15 min, incubated with antibodies for 20 min at 4 °C, and washed before sorting CD115+/CD11c-/NK- monocytes using an Influx cell sorter (BD) [68]. Monocytes were cultured in complete RPMI medium (Thermo Fisher Scientific) and exposed to 100 ng/mL CSF1, 50 µM Q-VD-OPh and 50 µM Ac-YVAD-cmk. After five days, macrophages were studied by flow cytometry using AlexaFluor700-F4/80 (#MCA497A700, Bio-Rad), BV510-CD11b (#101245, Biolegend), PerCP/Cy5.5-CD11c (#560584, BD), APC/Cy7-GR1 (#108424, Biolegend), PE-CF594-SiglecF (#562757, BD), PE-CD71 (#113808, Biolegend), PE/Cy7-CD206 (#141720, Biolegend), APC-CD54 (#116120, Biolegend), PB-CD40 (#124626, Biolegend), BV711-CD64 (#139311, Biolegend), BV605-IA-IE (#563413, BD), BUV737-CD43 (#564398, BD). For the sorting of monocytes, the antibodies used were the following: APC-CD11b (#17-0112-83, Thermo Fisher Scientific), PE/Cy7-CD11c (#561022, BD), FITC-NK (#553164, BD), PerCP/Cy5.5-Ly6C (#560525, BD), AlexaFluor700-Ly6G (#561236, BD), biotin-CD115 (#135507, Biolegend), PE-Streptavidin (#554061, BD). The data were analyzed with FlowJo software v. 10.0.00003.

### 4.19. Lung Fibrosis Model

Animals were injected intraperitoneally with bleomycin sulphate (0.1 mg/g body weight) once a week, for three weeks to induce lung fibrosis. Some of them were injected intraperitoneally with Z-VAD-FMK (1 µg/g body weight) every day or sub-cutaneously with IDN-6556 (18 µg/g body weight) twice a day, for three weeks. Animal procedures were performed according to the protocols approved by the Ethical Committee CEEA 26 and the French Ministry of Research #9861, in accordance with recommendations for the proper use and care of laboratory animals.

### 4.20. Fibrosis Quantification

To quantify the extent of collagen fibers, left lungs were fixed in 4% formaldehyde, paraffin embedded, cut into 4-µm sections, stained with Sirius Red, scanned using a microscopy virtual slide system (Olympus VS120), and analyzed using ImageJ 1.50b software. To quantify airspace number, tissue sections 4-µm stained with Sirius Red were scanned using a NanoZoomer-SQ (Hamamatsu Corporation, Shizuoka, Japan). Images of entire lung sections were recorded by means of NDP.view.2 software (Hamamatsu Corporation, Shizuoka, Japan) and analyzed at ×20 magnification with a pixel size of 0.452 µm. To quantify fibrosis, we used a numerical software program that allows a fully automatic selection of airspaces (alveoli and ducts) from the entire lung sections, without the large bronchi and vessels. Fibrosis severity was indicated by the ratio between the number of airspaces and the total area of parenchymal tissue.

### 4.21. Murine Interstitial Macrophage Collection and Analysis

Right lungs were digested with the Lung Dissociation kit (Miltenyi Biotec), filtered, erythrocytes were removed using ACK and nucleated cells were collected. Cells were washed with ice-cold PBS, incubated with Fc block (Murine TruStain FcX, Biolegend, 1/50 dilution) for 15 min, incubated with the antibodies for 20 min at 4 °C, washed, then fluorescence was measured with a BD LSRFortessa X-20. Antibodies used were the following: AlexaFluor700-F4/80 (#MCA497A700, Bio-Rad), FITC-CD45 (#103108, Biolegend), BV510-CD11b (#101245, Biolegend), PerCP/Cy5.5-CD11c (#560584, BD), APC/Cy7-GR1 (#108424, Biolegend), PE-CF594-SiglecF (#562757, BD), PE-CD71 (#113808, Biolegend), PE/Cy7-CD206 (#141720, Biolegend), APC-CD54 (#116120, Biolegend), PB-CD40 (#124626, Biolegend), BV711-CD64 (#139311, Biolegend), BV605-IA-IE (#563413, BD), BV650-CD24 (#563545, BD), BUV737-CD43 (#564398, BD). The data were analyzed with FlowJo software v. 10.0.00003. Interstitial macrophages were selected according to their larger size (FSC) and granularity (SSC), on CD45 expression to select leukocytes and GR1-positive cells will be excluded to remove neutrophils, they are equally CD11b high, SiglecF negative, IA-IE positive and CD24 negative.

### 4.22. Bronchoalveolar Lavage Fluid (BALF) Collection and Analysis

After cervical dislocation, the trachea was cannulated and secured using a silk suture. The right lung was ligated and PBS (300 µL) was slowly delivered in the left lung and retrieved through the cannula, the lavage was repeated two times. BALF were centrifuged at 600× *g* 10 min at 4 °C, and the acellular fraction stocked at −80 °C for future cytokine analysis. IL-2, IL-5, IL-6, KC, TNF-alpha and IL-1 beta were analyzed in the murine bronchoalveolar lavage fluids, using the Mouse Pro-Inflammatory Panel 1 V-Plex (MSD, Rockville, MD, US). The kit was run according to the manufacturer’s guidelines and the chemiluminescence signal was measured on a Sector Imager 2400 (MSD). To quantify TGF-beta1, a Milliplex TGF-beta1 Single Plex magnetic bead kit (Merck Millipore) was performed following the manufacturer’s instructions using the Bio-Plex200 system (Bio-Rad). The samples were not diluted (50 µL of BALF) for the assay.

### 4.23. Statistical Analysis

Data are means ± SE. Statistical significance was determined using Mann–Whitney test, Fisher Exact test, Fisher test with Bonferroni correction, Kruskal–Wallis test with Dunn’s post test or Two-way ANOVA with Bonferroni post test, as indicated. *p* < 0.05 was considered statistically significant.

## 5. Conclusions

The recruitment of functional macrophages in damaged tissues critically depends on monocyte response to CSF-1, which includes the activation of several caspases. In the present study, we depict a pathway in which caspase-7 is activated at the surface of mitochondria in monocytes exposed to CSF-1. In turn, caspase-7 cleaves p47*^PHOX^*, which activates NOX2 in phagocytes to generate superoxide anions and promote their migration capability. These results shed light on a specific, non-apoptotic function of caspase-7 in the generation of macrophages. Caspase inhibition prevents the development of bleomycin-induced lung fibrosis in mice, suggesting an alternative to CSF1R blockade and other strategies aiming to modulate tissue macrophage activity in various pathological settings.

## Data Availability

RNA sequencing data are in the ArrayExpress database at EMBL-EBI (www.ebi.ac.uk/arrayexpress) under accession number E-MTAB-5752 (Username: Reviewer_E-MTAB-5752; Password: y9PIG6yd).

## Supplementary Materials

The following supporting information can be downloaded at: https://www.mdpi.com/article/10.3390/ijms24044151/s1.
