# Supplementary material for: Caspase Inhibition Modulates Monocyte-Derived Macrophage Polarization in Damaged Tissues
Source: Int J Mol Sci. 2023 Feb 19;24(4):4151. doi: 10.3390/ijms24044151 (PMC9964254; doi:10.3390/ijms24044151)
Supplement: Supplementary file 1 [file ijms-24-04151-s001.zip › ijms-2135444-supplementary.pdf]

SUPPLEMENTAL

TITLE:

Caspase inhibition modulates monocyte-derived macrophage polarization in damaged tissues

Stéphanie Solier [1,2,3,*], Michele Mondini [4], Lydia Meziani [4], Arnaud Jacquel [5], Catherine Lacout [1,2], Tom Vanden Berghe [6,7], Yvon Julé [8], Jean-Claude Martinou [9], Gérard Pierron [10], Julie Rivière [1,2], Marc Deloger [11], Corinne Dupuy [12], Anny Slama-Schwok [12], Nathalie Droin [13,2], Peter Vandenabeele [6,7], Patrick Auberger [5], Eric Deutsch [4], Jamel El-Benna [14], Pham My-Chan Dang [14] and Eric Solary [13,2]

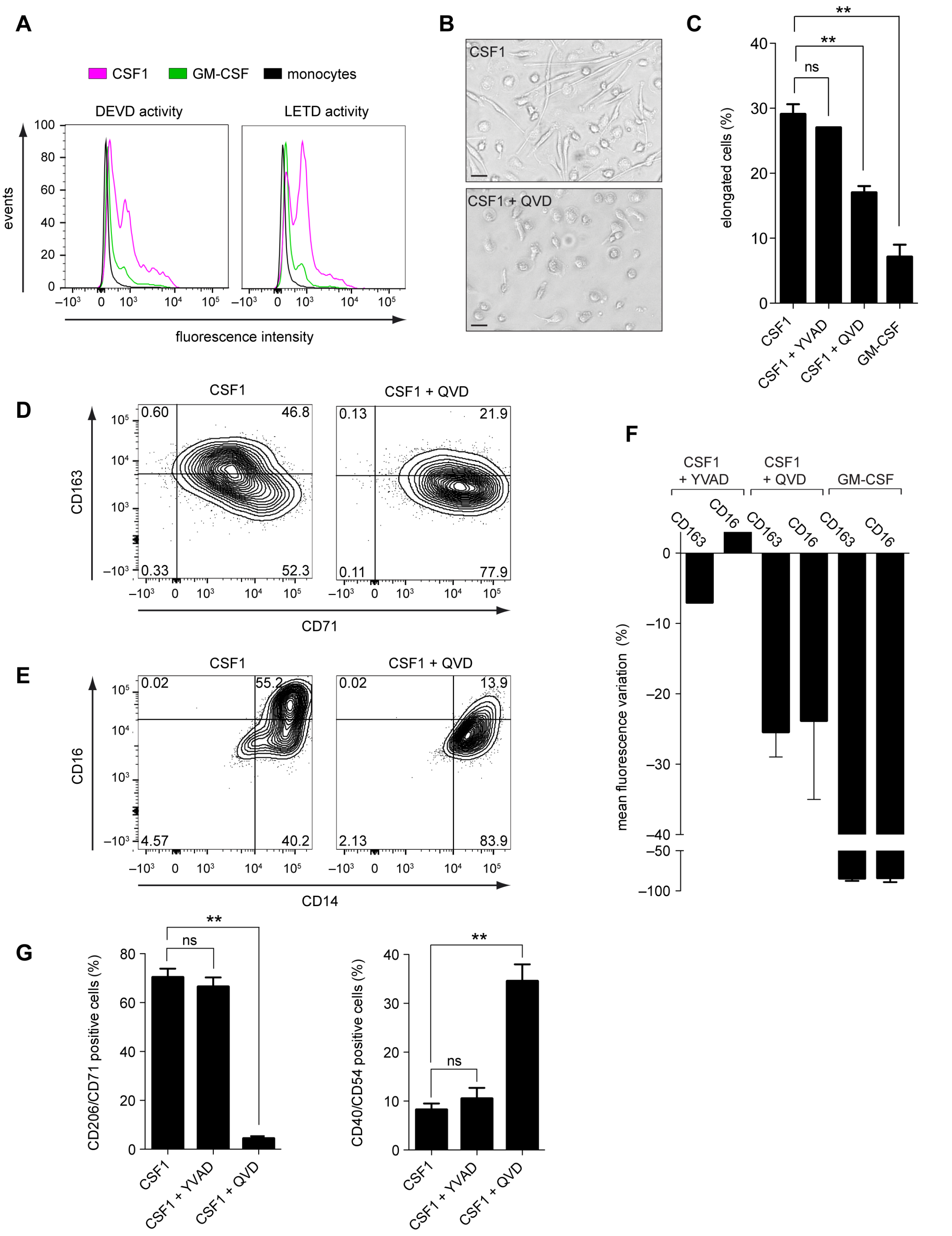

Figure S1. Caspase inhibition impacts macrophagic differentiation. (A) Human CD14$^+$ monocytes were treated with 100 ng/mL CSF1 or 100 ng/mL GM-CSF for 24 hours before measuring FAM-DEVD-FMK or FAM-LETD-FMK fluorescence by flow cytometry. **(B)** Human peripheral blood CD14$^+$ monocytes acquire a characteristic "fibroblast-like" elongated shape when treated with 100 ng/mL CSF1 for 4 days (upper panel), which is prevented by co-culture with 50 µM Q-VD-OPh (QVD) (lower panel). Representative microscopy pictures under visible light. Scale bars: 20 µm. **(C)** The fraction of elongated cells (elongation factor >

2.5) was measured after 4 days of monocyte treatment with 100 ng/mL CSF1 alone, 100 ng/mL CSF1 and 50 μM Ac-YVAD-cmk (YVAD), 100 ng/mL CSF1 and 50 μM Q-VD-OPh (QVD), and 100 ng/mL GM-CSF. Mean ± SE of three independent experiments (***, P<0.001/6; ns, non significant; Fisher test with Bonferroni correction). **(D)** Representative scatter plots showing the distribution of CD163 and CD71 in CSF1-treated monocytes and CSF1+QVD-treated monocytes. **(E)** Representative scatter plots showing the distribution of CD16 and CD14 in CSF1-treated monocytes and CSF1+QVD-treated monocytes. **(F)** Flow cytometry analysis of CD163 and CD16 at the surface of monocytes treated with CSF1 and YVAD or CSF1 and QVD or GM-CSF as above. Results are expressed as the variations in mean fluorescence intensity compared to CSF1-treated monocytes. Mean ± SE of at least three independent experiments. **(G)** Caspase inhibition also affects murine monocyte differentiation, i.e. mouse bone marrow cells were flushed from femurs and tibias, $CD115^+$/ $CD11c^-$ /NK^- cells were sorted and treated with 100 ng/mL CSF1 alone or with 50 μM QVD, and 50 μM YVAD for 5 days before measuring the percentage of $CD206^+$/$CD71^+$ positive and $CD40^+$/$CD54^+$ positive cells (**, P<0.01; ns, non significant; Mann-Whithney test, n=6). Mean ± SE.

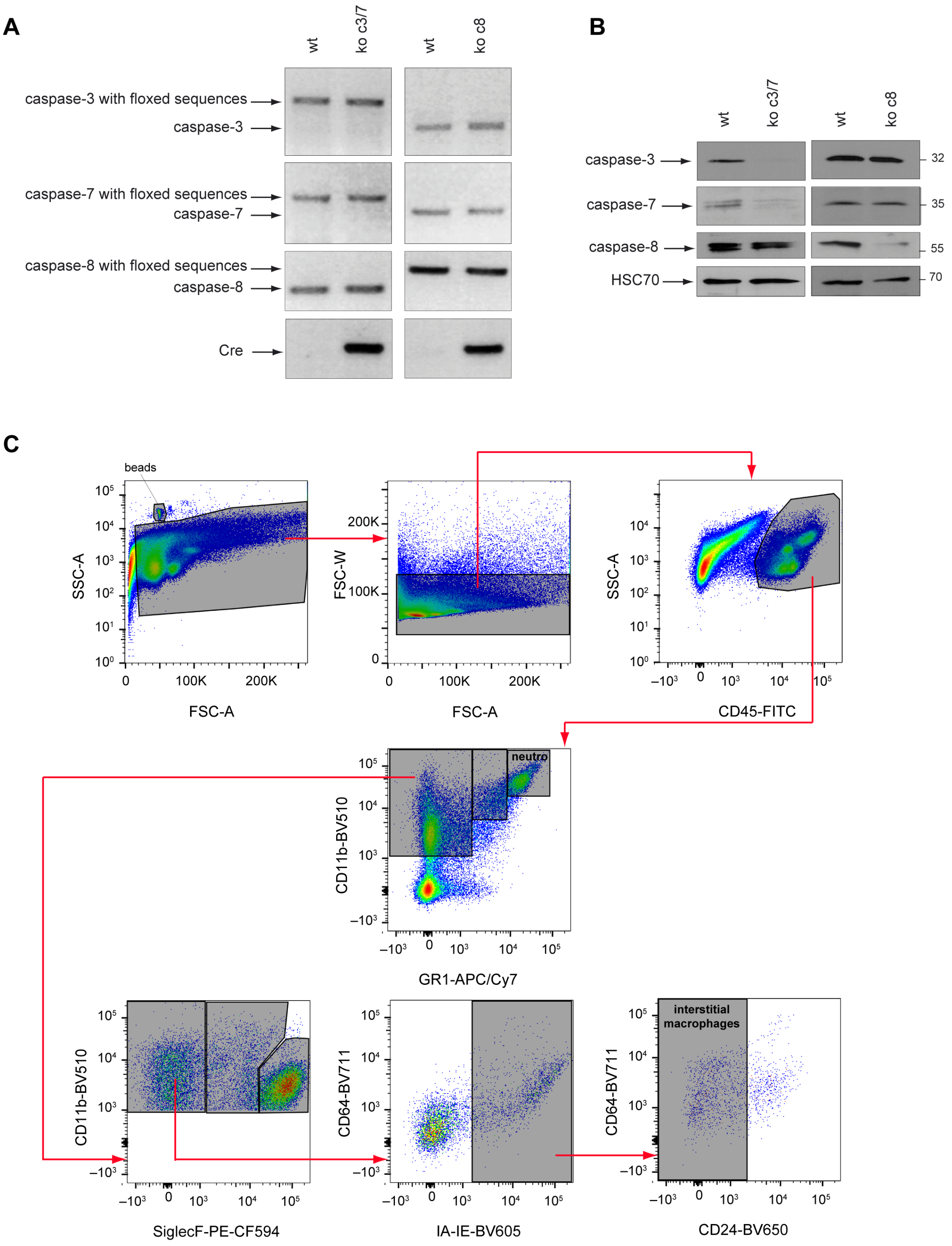

Figure S2. Genotyping of the murine models and gating strategy used to FACS-analyze murine interstitial macrophages. (A) PCR analysis of mouse tail DNA with caspase-3, caspase-7 and caspase-8 primer sets flanking the floxed sequences. The PCR showed the persistent expression of floxed genes in non-monocytic cells of wild-type and knockout mice. The Cre status was checked using Cre primers. **(B)** Immunoblot expression of indicated caspases in sorted mouse monocytes with various genotypes treated for 5 days with CSF1.

HSC70, loading control. The residual bands may be related to the contamination of enriched mouse monocytes with non monocytic / granulocytic cells or to incomplete efficacy of the recombinase. Protein ladder (kDa) is indicated. (**C**) Interstitial macrophages were selected according to their larger size (FSC) and granularity (SSC) as $CD45^+$, $GR1^-$, $CD11b^{high}$, $SiglecF^-$, $IA\text{-}IE^+$, $CD24^-$ cells.

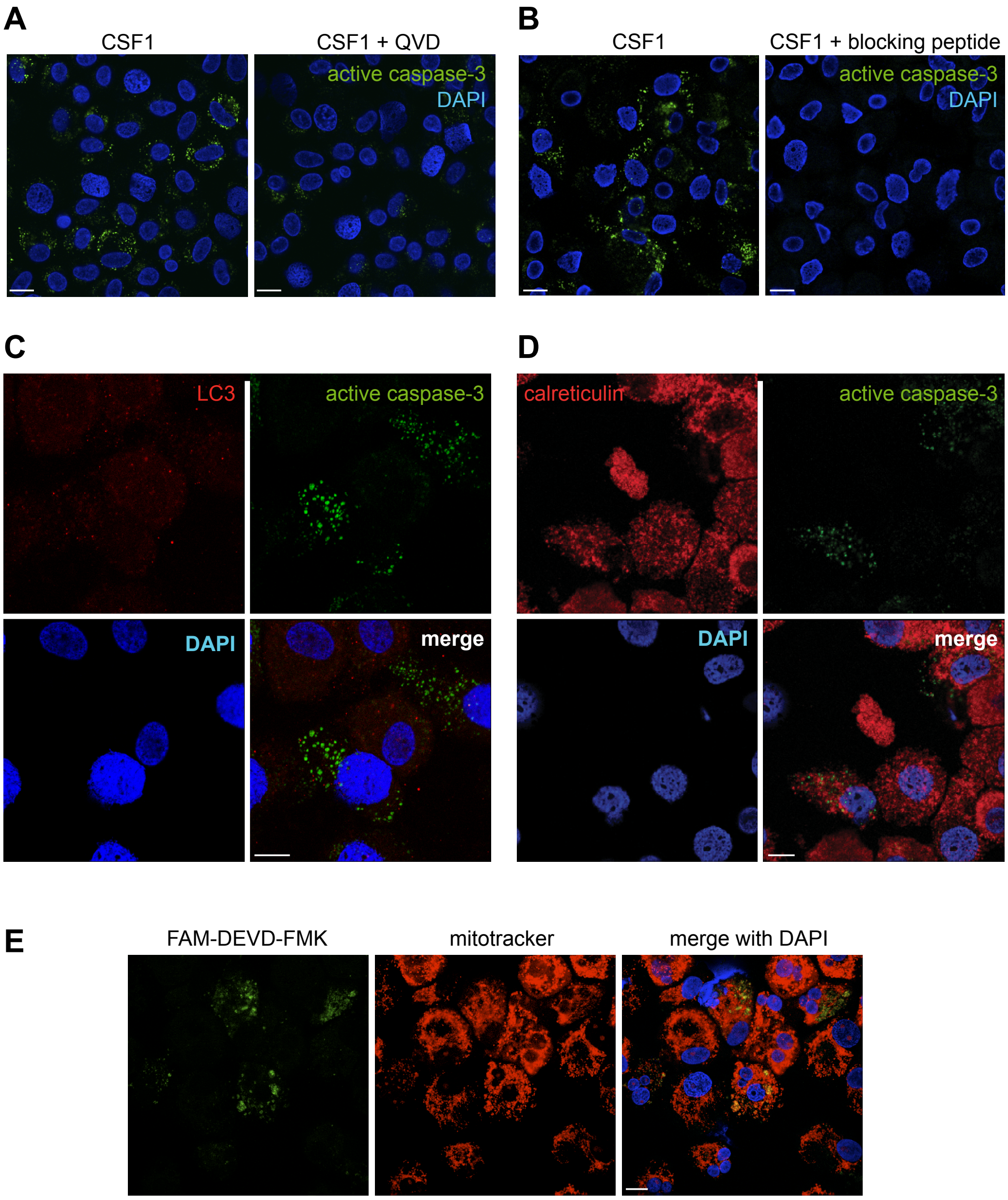

Figure S3. Mitochondrial active caspase-3 in CSF1-treated monocytes. Confocal immunofluorescence analyses. In all figures, nuclei were stained in blue with DAPI. Scale bars: 10 µm. **(A)** Human peripheral blood monocytes were treated for 4 days with CSF1 alone or combined with Q-VD-Oph (QVD) (as in other expreriments), then labeled with an anti-active caspase-3 antibody (green). Mean of the green fluorescence: 188 (CSF1); 129 (CSF1+QVD). **(B)** CSF1-treated monocytes were labeled with an anti-active caspase-3 antibody (green) in the absence or presence of a blocking peptide. Mean of the green fluorescence: 134 (CSF1); 66 (CSF1+blocking peptide). **(C)** CSF1-treated monocytes labeling with an anti-active caspase-3 antibody (green) and an anti-LC3 antibody (red) did not detect any colocalization with autophagic granules. Threshold set to 300 (green) and 1000 (red) for co-localization analyses with ImageJ-JACoP; Manders coefficient: 0.12 (fraction of active caspase-3 co-localized with LC3). Manders coefficient is low demonstrating the

absence of colocalization. **(D)** CSF1-treated monocytes labeling with an anti-active caspase-3 antibody (green) and an anti-calreticulin antibody (red) did not detect any colocalization with endoplasmic reticulum. Threshold set to 300 (green) and 2500 (red) for co-localization analyses with ImageJ-JACoP; Manders coefficient: 0.16 (fraction of active caspase-3 co-localized with endoplasmic reticulum). Manders coefficient is low demonstrating the absence of colocalization. **(E)** DEVD activity (green) and mitochondria (mitotraker, red) co-staining in CSF1-treated monocytes.

Solier S et al, Supplemental Figure 4

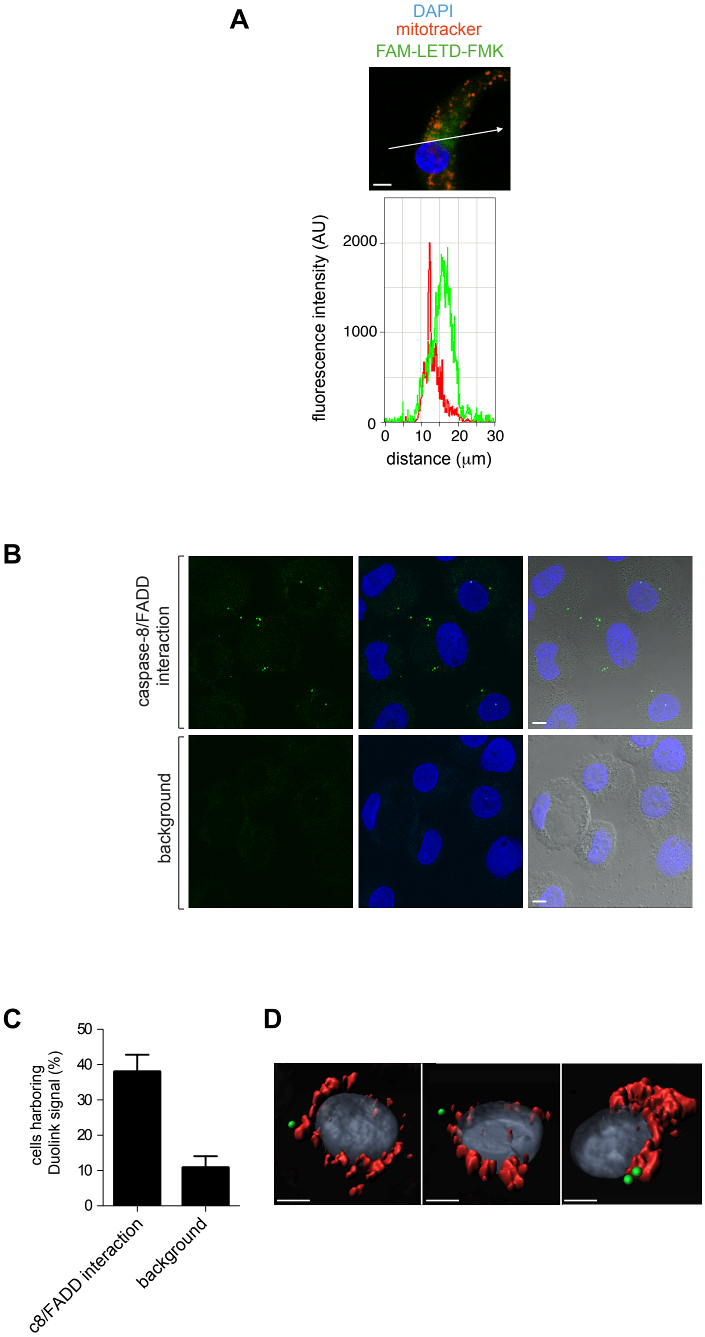

Figure S4. Subcellular localization of active caspases-8 in CSF-treated monocytes. (A) Confocal microscopy image (upper panel) and intensity tracing (lower panel) showing the distribution of FAM-LETD-FMK (green) and mitotracker (red) in a CSF1-treated monocyte. The nucleus was stained in blue with DAPI. Scale bar, 5 µm. **(B)** Duolink *in situ* proximity ligation assay looking for interactions between caspase-8 and FADD (green) in CSF-1-treated monocytes. Nuclei were stained in blue with DAPI. Right panels: overlay of fluorescence and visible light microscopy images. Scale bar, 5 µm. **(C)** Quantification of the duolink *in situ* proximity ligation assay for interactions between caspase-8 and FADD, indicating the percentage of CSF-1-treated monocytes harboring a Duolink signal. Mean ± SE. **(D)** 3D-representation of CSF-1-treated monocytes showing caspase-8/FADD interaction in green, mitochondria in red (mitotracker), and nuclei in gray (DAPI). Scale bar, 5 µm.

Solier S et al, Supplemental Figure 5

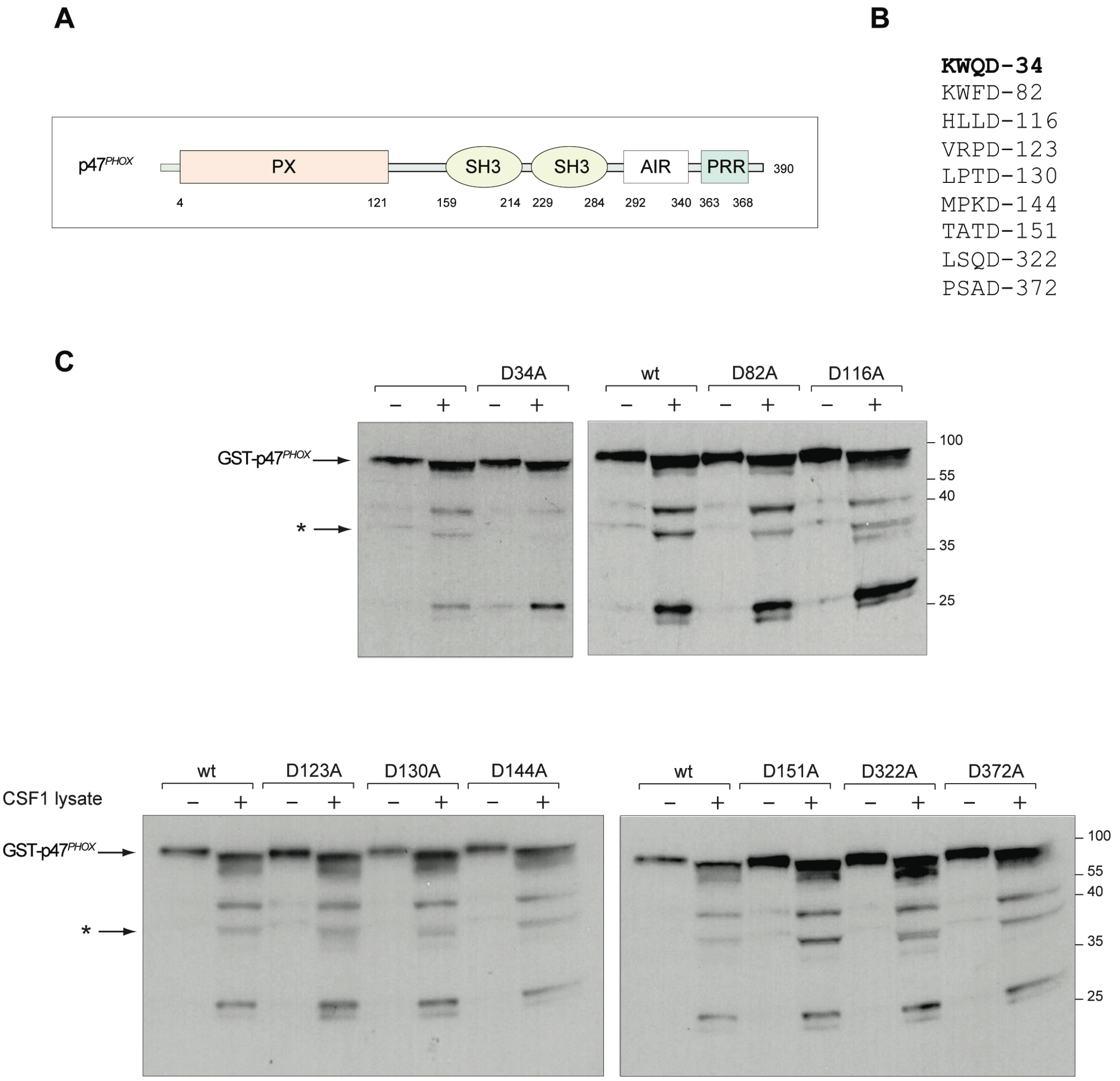

Figure S5. Caspase-dependent cleavage of p47^{PHOX} in CSF1-treated monocytes. (A) Schematic representation of p47^{PHOX} domains. p47^{PHOX} is composed of 390 amino acids with a phox-homology (PX) domain, two src-homology-3 domains (SH3), an autoinhibitory region (AIR) and a proline-rich region (PRR). **(B)** Potential caspase cleavage sites in p47^{PHOX} sequence that were tested by using an *in vitro* caspase cleavage assay. The P4-P1 amino acid sequence and the position of the aspartate (D) in P1 that was replaced by an alanine (A) are indicated. Except for D34A (in bold), all the other mutations had no impact on caspase-mediated cleavage of p47^{PHOX}. **(C)** Wild-type and mutated p47^{PHOX} were expressed in *E. coli* and the proteins were incubated with CSF1-treated monocyte lysates (80 µg) before analyzing GST- p47^{PHOX} cleavage by immunoblotting using an anti-GST antibody. Black star, cleavage fragment. "D34A panel" and Figure 6E are independent experiments showing similar results. Protein ladder (kDa) is indicated.

Solier S et al, Supplemental Figure 6

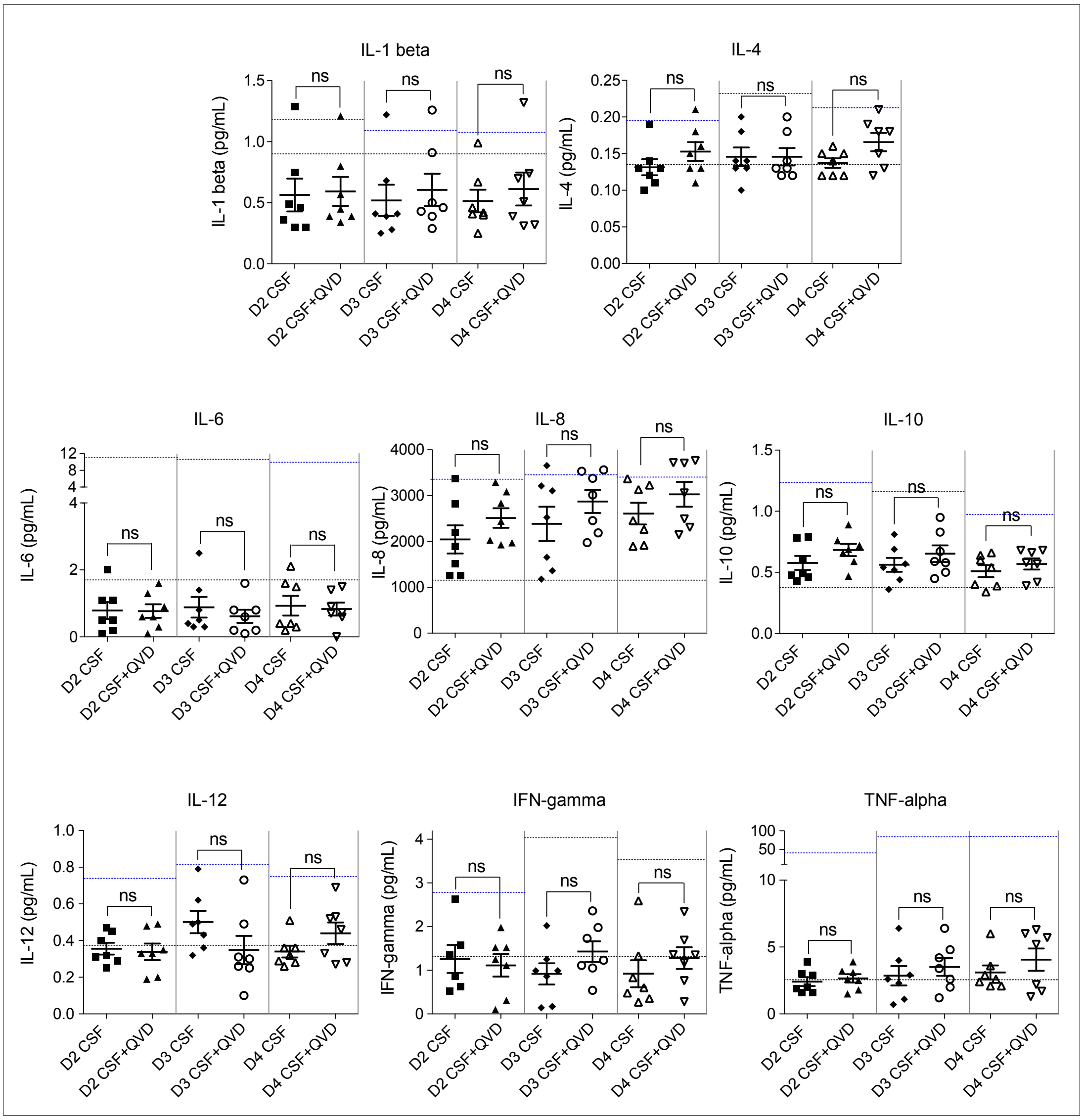

Figure S6. Caspase inhibition does not change the cytokinic profile of CSF1-treated monocytes. Inflammatory cytokines were measured in culture supernatants of human monocytes treated by CSF1 or CSF1+QVD during 2, 3 and 4 days. Results were expressed in pg/mL. (ns, non significant; Mann-Whithney test). The black dashed line corresponded to cytokine concentration in untreated monocytes, the blue dashed line to cytokine concentration in GM-CSF-treated monocytes.

Solier S et al, Supplemental Figure 7

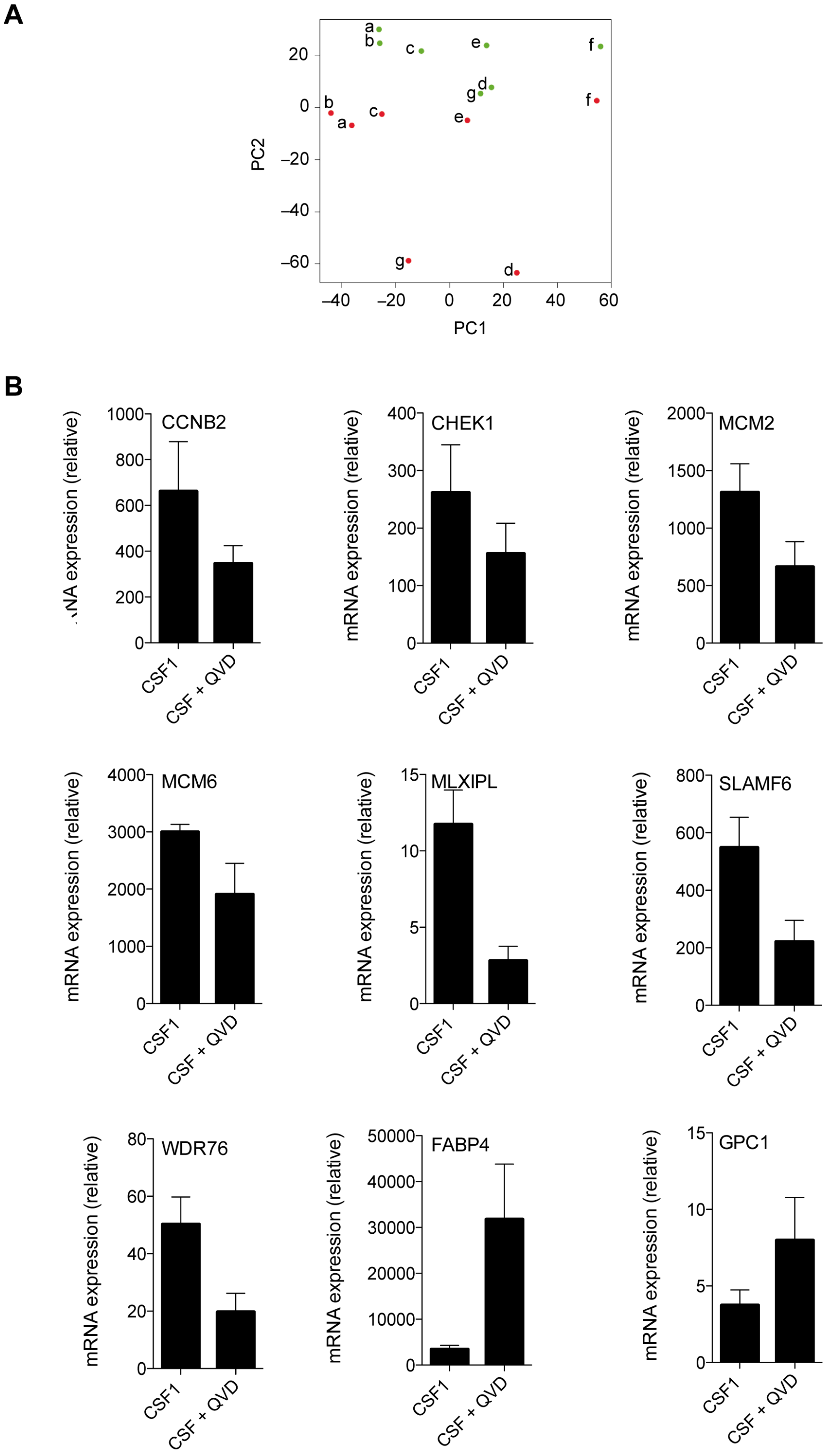

Figure S7. Gene expression variations detected by RNA sequencing. (A) Principal component analysis of RNA sequencing data showing CSF1-treated samples as green circles, and CSF1 + QVD treated samples as red circles. Individual blood donors are identified by a letter. **(B)** qRT-PCR measurement of the expression of 9 genes identified as significantly modified by co-treatment of CSF-1-treated monocytes with Q-VD-OPh (QVD) (Mean ± SE of triplicates).

Supplemental Table S1: Oligonucleotides used for mutagenesis (5' → 3', F: forward, R: reverse)

Mutation	F/R	Oligonucleotide sequences
D34A	F	GAAATGGCAGgccCTGTCGGAGA
	R	ACCAGGAACATGTACACATAGTG
D82A	F	CAAGTGGTTTgccGGGCAGCGGG
	R	GGAGCTGGGAGGTGGGGG
D116A	F	CCACCTCCTCgccTTCTTCAAGG
	R	GGACAGCGGGAGATCTTG
D123A	F	GGTGCGCCCTgctGACCTCAAGC
	R	TTGAAGAAGTCGAGGAGGTGGG
D130A	F	GCTCCCCACGgccAACCAGACAA
	R	TTGAGGTCATCAGGGCGC
D144A	F	GATGCCCAAAgctGGCAAGAGTA
	R	AAGTATGTCTCTGGCTTTTTTG
D151A	F	TACCGCGACAgccATCACCGGCC
	R	CTCTTGCCATCTTTGGGCATCAAGTATG
D322A	F	CCTCAGCCAGgccGCCTATCGCC
	R	CGCTTCCGCGACCGCTGATG
D372A	F	GCCGAGCGCCgccCTCATCCTGAACCGC
	R	CGCGGGGGCACCGCCGGC
p47^{PHOX} trunc 82	F	GGGCAGCGGGCCGCCGAG
	R	CAGGGGCCCCTGGAACAGAACTTCCAG

Supplemental Table S2. Primers used for qRT-PCR to validate RNA Seq data (5' → 3', F: forward, R: reverse)

Gene name	F/R	Primer sequences
CCNB2	F	TGCAAAATCGAGGACATTGA
	R	TGTGGGTTTATGGACTGCAA
CHEK1	F	CTGAAGAAGCAGTCGCAGTG
	R	TTGCCTTCTCTCCTGTGACC
FABP4	F	CATCAGTGTGAATGGGGATG
	R	CTGGCCCAGTATGAAGGAAA
GPC1	F	CCTGTCAGAGCAGGAAGGAC
	R	GTAAGGGCCAGGAAGAGGAG
HPRT	F	GGACAGGACTGAACGTCTTGC
	R	CTTGAGCACACAGAGGGCTACA
L32	F	TGTCCTGAATGTGGTCACCTGA
	R	CTGCAGTCTCCTTGCACACCT
MCM2	F	TTGCTGTAGGGGAACTGACC
	R	TCTCCGATCTGCTGATCCTT
MCM6	F	GCTTGTGAGCGGAACTTTTC
	R	CTGGATTTCGGCAGATGTTT
MLXIPL	F	TACAGTGGCAAGCTGGTGTC
	R	TTCAGGCGGATCTTGTCTCT
PPIA	F	GTCGACGGCGAGCCC
	R	TCTTTGGGACCTTGTCTGCAA
SLAMF6	F	TGACCTGTGAGCTCCATCTG
	R	TCCCAGGAGACAGTGAGGTT
WDR76 F	F	GGATAGACGGACACCTGGAA
	R	CACTGGGTGGACATGAACAG

Supplemental Table S3. Primers used for mice genotyping (5' → 3', F: forward, R: reverse)

Gene name	F/R	Primer sequences
Casp 3	F	TTTTGAGGTGGAGTTTTGC
	R	TCTACTGCTTTTACTCTCTTCC
Casp 7	F	TCGATTGATGGCACGTTCTAGC
	R1	CCACGAGGAATAAGTGGTCAC
	R2	GGAATTTGGAGCTTGCCAACC
Casp 8	F	ATAATTCCCCCAAATCCTCGCATC
	R	GGCTCACTCCCAGGGCTTCCT
Cre	F	GCCTGCATTACCGGTCGATGCAACGA
	R	GTGGCAGATGGCGCGGCAACACCATT
